# Heterogeneity of Gene Expression in Murine Squamous Cell Carcinoma Development—The Same Tumor by Different Means

**DOI:** 10.1371/journal.pone.0057748

**Published:** 2013-03-18

**Authors:** Noam Cohen, Nataly Kravchenko-Balasha, Shoshana Klein, Alexander Levitzki

**Affiliations:** Unit of Cellular Signaling, Department of Biological Chemistry, The Alexander Silberman Institute of Life Sciences, The Hebrew University of Jerusalem, Jerusalem, Israel; Kyushu Institute of Technology, Japan

## Abstract

Transformation is a complex process, involving many changes in the cell. In this work, we investigated the transcriptional changes that arose during the development of squamous cell carcinoma (SCC) in mice. Using microarray analysis, we looked at gene expression during different stages in cancer progression in 31 mice. By analyzing tumor progression in each mouse separately, we were able to define the global changes that were common to all 31 mice, as well as significant changes that occurred in fewer individuals. We found that different genes can contribute to the tumorigenic process in different mice, and that there are many ways to acquire the malignant properties defined by Hanahan and Weinberg as “hallmarks of cancer”. Eventually, however, all these changes lead to a very similar cancerous phenotype. The finding that gene expression is strongly heterogeneous in tumors that were induced by a standardized protocol in closely related mice underscores the need for molecular characterization of human tumors and personalized therapy.

## Introduction

Cancer is a collection of more than 100 different diseases, and each of these diseases consists of several variants that can develop differently in different individuals. Tumorigenesis occurs due to changes in the biochemical networks and signaling networks that drive the normal cell. With time the cell accumulates mutations and epigenetic changes, which alter the signaling and biochemical networks, and can lead to cell transformation and cancer [1]. Although there are a few cases in which a disease can be linked to one major signaling event (e.g. Bcr-Abl in CML [2]), in most tumors this is not the case. Genetic, epigenetic and environmental perturbations occur throughout tumor development. Usually, the tumor is dependent on several oncogenic signals. Furthermore, the intrinsic genomic instability of cancer cells leads to continual evolution and to intra-tumor heterogeneity [3].

The microarray technology has become a popular and common strategy to study gene regulation in cancer [4]–[7]. Although gene expression can also be regulated at the level of DNA, by mutation or epigenetic modifications, as well as post-transcriptionally, mRNA levels are considered a legitimate measure of gene expression, and analysis of expression microarrays is a valid method for analysis of changes in cellular functions. There are several ways to analyze microarray data, as described in [8]–[10]. One of the main hurdles in microarray analysis is the heterogeneity between biological replicates. In most cases, the analyst attempts to smooth over the heterogeneity, and looks at averaged expression changes that are significant in most or all of the replicates [11], [12]. Cluster analysis then delineates groups with significant differences. Although for many purposes this average analysis is appropriate, heterogenic data reflect real differences between biological replicates. These differences, which are minimized when looking at average expression, can have profound phenotypic effects.

In recent years, the concept of personalized therapy has gained popularity [13]–[15]. Two fundamental principles that underlie the concept of personalized cancer therapy are that significant genomic heterogeneity exists among tumors, even those derived from the same tissue of origin, and that these differences can play an important role in determining the likelihood of a clinical response to treatment with particular agents. Such genomic heterogeneity can involve differences in the spectrum of coding sequence mutations, as well as focal gene amplifications, deletions, or translocations. It might also involve epigenetic changes in the expression profile of a tumor cell, although the sources of epigenetic variation among tumors remain poorly understood [16].

In this study, we have looked at tumor heterogeneity in mice of similar genetic background. These mice shared the same living conditions and were treated with the same carcinogens, and all developed squamous cell carcinoma. We compared the results of averaging microarray data with the results of analyzing each tumor on a case-by-case basis. The case-by-case analysis highlighted the surprising degree of heterogeneity of oncogenic signaling between the mice.

## Materials and Methods

As described by Quigley et al., male SPRET/Ei mice were mated with female FVB/N mice, and the female F1 hybrids were back-crossed to FVB/N males. Skin tumors were induced on dorsal back skin of the resulting FVBBX mice by treatment with dimethyl benzanthracene (DMBA) and tetradecanoyl-phorbol acetate (TPA). Multiple benign papillomas and malignant squamous cell carcinomas (SCC) developed. Normal tail skin, papillomas and carcinomas were harvested when mice were sacrificed due to presence of a carcinoma and microarray analysis was performed. Microarray data used in the current analysis were from GEO (GSE21264). 31 mice were analyzed; for each of these mice we have data for all 3 progression steps (normal, papilloma, and carcinoma). The mouse ID numbers were the same IDs as in Quigley et al. [17] Genes were selected for analysis based on detection and fold change. The starting data set represented 45,101 probe sets. The expression value of every gene in papilloma (P) and carcinoma (C) was normalized to the average expression value of the same gene in normal (N), and the resulting ratios were transformed to log2. For the average analysis, T-test was then invoked, comparing each of P and C cells to N, and only genes with p value<0.05 were analyzed further (significant genes). Genes showing greater than 4-fold change were assigned to functional groups using DAVID software and KEGG database. Overrepresented gene categories were identified using DAVID software. Results were filtered to remove categories with EASE score (a conservative variant of the one-tailed Fisher's exact probability) more than 0.001 and FDR>0.

Pathways showing greater than 4-fold change according to DAVID were detected by the KEGG database.

Data were analyzed in 2 ways:

1. “Average analysis”: For each transcript on the microarray, the expression over all 31 mice at each stage (normal, papilloma and carcinoma) was averaged. Ratios of expression between the stages, i.e. papilloma/normal (P/N), carcinoma/papilloma (C/P), carcinoma/normal (C/N) were calculated and filtered to select transcripts that showed at least a 4-fold change and had a P-value≤0.05 in a T-test, to get a list of transcripts with significant changes.
2. “Heterogeneity analysis”: To compare the changes in gene expression during cancer progression between different individuals, we analyzed each mouse separately, using the expression data for normal skin, papilloma and carcinoma from that specific mouse. For each transcript, P/N, C/P and C/N were calculated and those transcripts that had at least a 4-fold change were chosen for further analysis.

### DAVID analysis

The lists of genes with 4-fold change were inserted into DAVID for annotation analysis. Only DAVID annotations that had P-values equal to or less than 0.001 were considered further. The annotations lists for the individual mice were analyzed using an algorithm that enabled us to examine which annotations were common to two or more mice and which were unique.

### KEGG analysis

For each mouse, we fed into KEGG a list of all the transcripts that were significantly changed between carcinoma and normal and searched for pathways that were altered in all or many of the mice. We then looked at the gene list in each category and asked which genes appeared in all of the mice.

## Results

### Heterogeneity in transcriptional regulation among carcinomas from different mice

**Figure 1:**
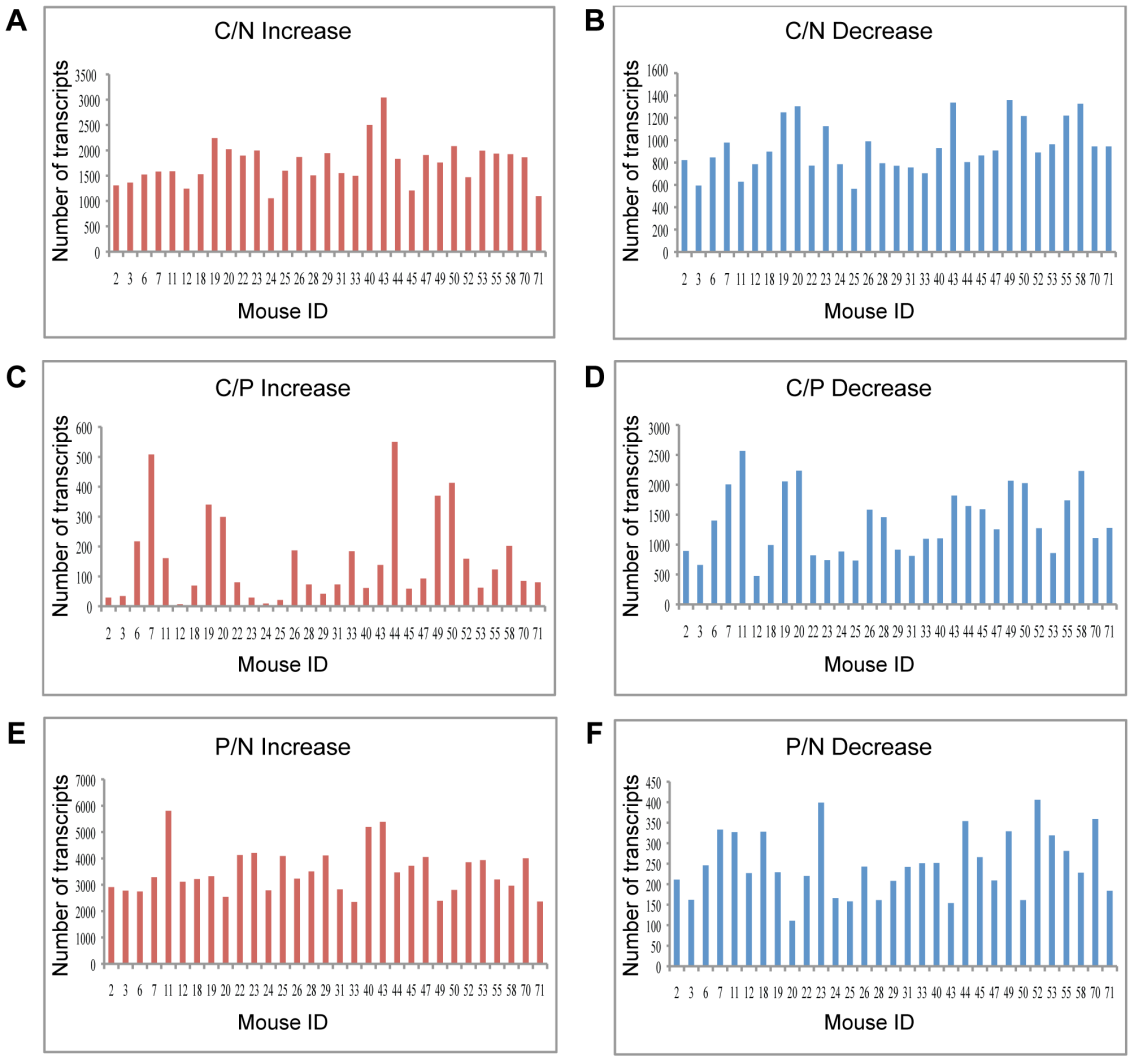
Analysis of individual mice reveals diversity in transcript number that changed during carcinogenesis. The number of transcripts that were up-regulated (left panel) or down-regulated (right panel) at least 4-fold in each mouse during the transition from: A. normal skin to carcinoma (C/N); B. papilloma to carcinoma (C/P); and C. normal skin to papilloma (P/N). Mouse IDs refer to the IDs in the original data [17].

Quigley et al. [17] induced skin tumors on dorsal back skin of mice from a Mus spretus/Mus musculus backcross ([SPRET/Ei×FVB/N]×FVB/N) using dimethyl benzanthracene (DMBA) and tetradecanoyl-phorbol acetate (TPA). This treatment induced multiple benign papillomas as well as malignant squamous cell carcinomas (SCC) and spindle cell carcinomas. Quigley et al. deposited Affymetrix Mouse Genome 430 2.0 array data from normal tail, papilloma and carcinoma for 31 mice. To obtain an overall picture of transcription variation among these 31 mice, we first asked how many transcripts were induced or repressed 4-fold in each mouse. A 4-fold (rather than the more usual 2-fold) cutoff was chosen, in order to minimize heterogeneity between mice. Even so, there was a marked variation between the mice in the numbers of transcripts that were up-regulated/down-regulated at each progression step (**Figure 1**).

### DAVID annotations vary among mice

We asked whether this mouse-to-mouse variation in transcription reflects the pathways that change during carcinogenesis in each mouse. To this end, significant genes were annotated using DAVID, and we examined which annotations were common to multiple mice and which were unique.

**Figure 2:**
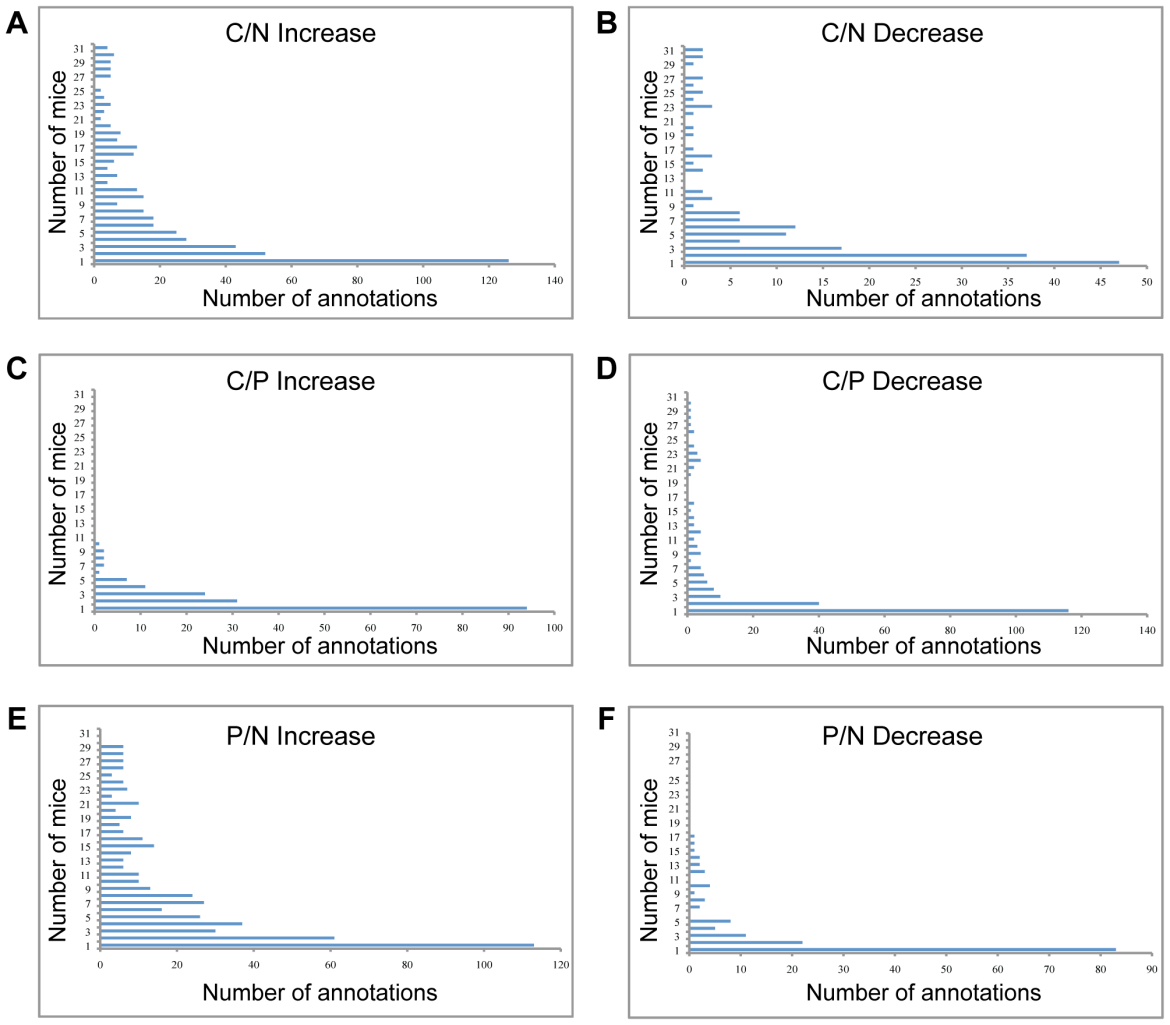
Heterogeneity in DAVID analysis of individual mice. The Y axis represents the number of mice in the group, and the X axis represents the number of significant annotations that were increased (left panel) or decreased (right panel) in each group. The annotations were examined in: (A) carcinomas vs. normal skin (C/N); (B) carcinomas vs. papillomas (C/P), and (C) papillomas vs. normal skin (P/N). Only 4 annotations were increased in C/N in all 31 mice, while 126 annotations were each increased in only one mouse.

Few annotations were significant in all 31 mice: only four annotations were up-regulated at least four-fold in all 31 carcinomas compared to normal skin (C/N) and two annotations were down-regulated (**Figure 2** **and Table S1A and S1B**). The categories of cell migration and cell motility (as well as the near-synonymous terms cell localization and cell motion) were up-regulated between C/N in all 31 mice. Increased migration and motility are characteristic of epithelial to mesenchymal transition (EMT) and increased invasiveness [18], [19]. We note that in some mice the categories of cell migration and cell motility were induced by the papilloma stage (P/N), whereas in others they were significantly induced upon progression from papilloma to carcinoma (C/P). As expected for categories that were over-represented in all 31 mice, these categories were also over-represented in the average analysis. Taken together, the data suggest that carcinoma development in all of the mice was dependent on EMT-like processes.

The annotations “fatty acid metabolic process” and “oxidation reduction” showed at least a four-fold decrease in all 31 mice (C/N). Upon more careful analysis, we found that the fatty acid processes that were decreased were mainly catabolic processes, presumably reflecting the fact that proliferating cells require fatty acids for membrane assembly. Indeed, the annotation “fatty acid catabolic processes” was down-regulated in most of the mice, albeit with a P-value that did not reach our stringent cutoff of 0.001.

Most of the DAVID categories appeared in only a minority of mice. In the comparison of carcinoma to normal skin, 126 categories were increased in expression at least four-fold in one mouse only, and 47 were decreased. Eighty-two percent of the categories that were increased at least four-fold were significant in less than half of the mice: 381 categories were increased in 15 or fewer mice, out of 466 categories that were increased in any number of mice. Eighty-eight percent of the categories that were decreased at least four-fold were significant in less than half of the mice: 151 categories were decreased, out of 172 categories in any number of mice (**Figure 2**). The variability in significant pathways identified by DAVID implies that different mechanisms can contribute to cancer progression in these mice, and that the features that characterize a cancer cell can arise from different molecular events.

### Heterogeneity in regulation within DAVID annotations

To further investigate the differences between the mice, we focused on a few specific annotations. Even when looking only at those mice in which DAVID designated a pathway as significant, the mode of regulation of the given category was different for different mice.

#### Cell death

In the average analysis, programmed cell death was not over-represented in DAVID. In the analysis of the individual mice, the category “regulation of programmed cell death” (which includes both positive and negative regulation) was over-represented in 18 mice. The category “negative regulation of cell death” was over-represented in DAVID, but only in 6 mice. (In two of these mice, the category “positive regulation of cell death” was also increased (see below)). The complementary category, “positive regulation of cell death”, was not down-regulated in any of the mice. These data imply that decreased apoptosis is not a prerequisite for SCC formation in this model.

Surprisingly, the category “positive regulation of cell death” was up-regulated in 10 carcinomas; in two of these, “negative regulation of cell death” was also up-regulated, so these processes may have balanced each other out. Similarly, in four mice, the DAVID annotation “regulation of programmed cell death” was significant, but neither “negative regulation of cell death” nor “positive regulation of cell death” was displayed, because a small number of genes of either type was induced, and the P-values for the subclasses “negative regulation of cell death” and “positive regulation of cell death” were greater than 0.001.

**Figure 3:**
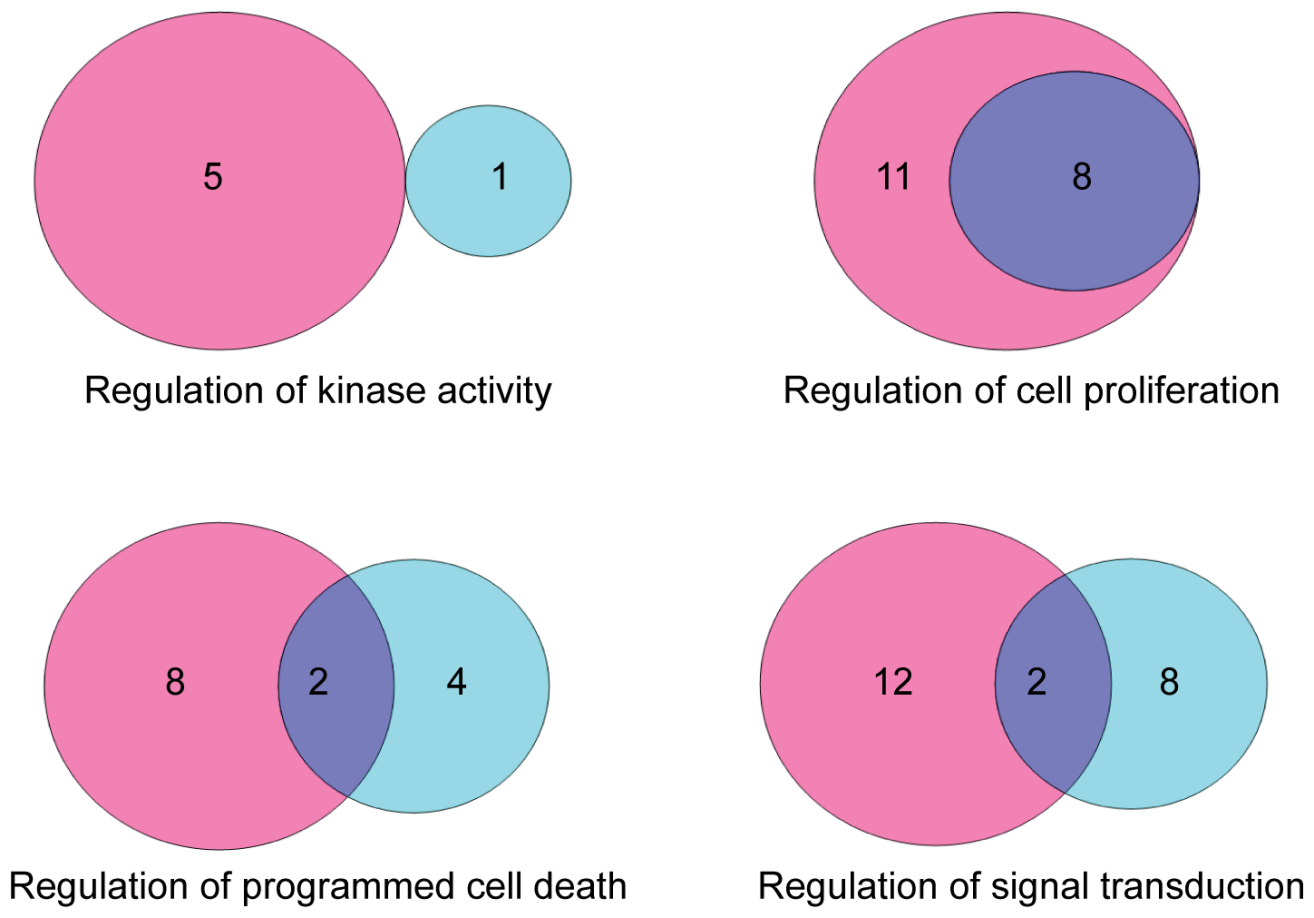
Differential regulation of specific annotations in DAVID. Selected annotations were analyzed to examine the manner in which the specific pathway is regulated. Venn diagrams show the number of mice in which the pathway showed negative (cyan), positive (pink), or both negative and positive regulation (purple).

In 8 mice there was a clear increase in “positive regulation of cell death”. In these 8 mice, the apoptosis signal was apparently turned on in the developing tumors, and nonetheless the tumors progressed into carcinomas. This finding implies that other cancer-promoting pathways were dominant, overcoming the increased apoptotic potential of these tumor (**Figure 3**).

#### Signal transduction

Regulation of signal transduction was significant in 22 mice. (Actually, DAVID does not have a category “regulation of signal transduction”; rather there are two annotations “positive regulation of signal transduction” and “negative regulation of signal transduction”.) In 55% (12/22) of the mice the category “positive regulation of signal transduction” was significant; in 9% (2/22) the category “negative regulation of signal transduction” was significant, and in 36% (8/22) of the mice both categories were significant, meaning that the regulation of signal transduction was both positive and negative. These data are not unexpected, since signal transduction includes oncogenic and tumor suppressive processes.

In summary, in the analysis of the individual mice, it became clear that even annotations with a clear relevance to cancer were significant in only some of the mice. Moreover, even among the mice for which a given annotation was significant, some mice showed overall positive regulation of the annotation, whereas others showed negative regulation of the same annotation. This compounds further the finding of heterogeneity between individual mice with the same tumors.

### Differences between “average analysis” and “heterogeneity analysis”

**Figure 4:**
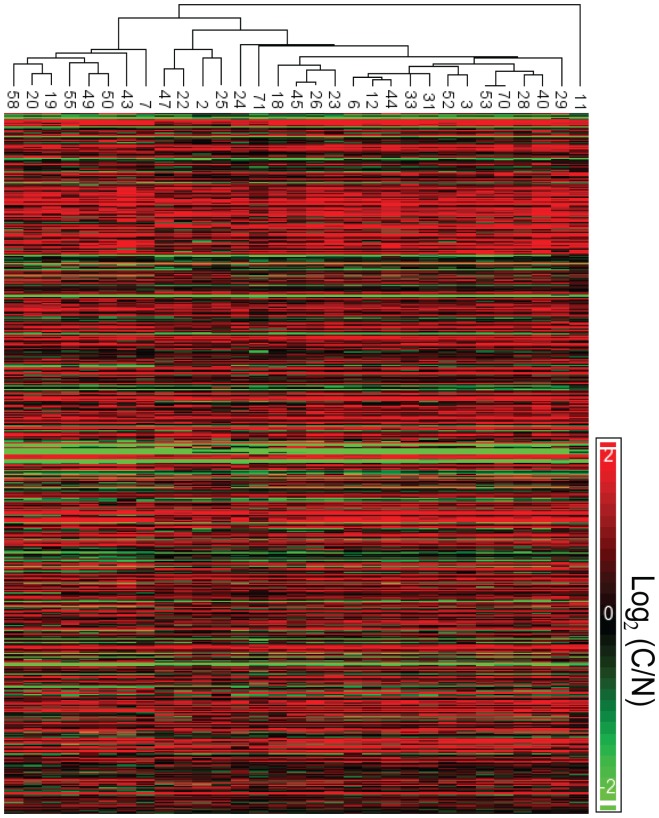
Heatmaps reveal heterogeneity between all 31 mice. Transcripts that were changed at least four-fold according to the average analysis are displayed with their expression level in each mouse separately. Although the mice can be clustered into closely related groups, no two mice show exactly the same expression pattern.

In the “average analysis” we took into account all the biological replicates of the same time point (e.g. carcinoma) and averaged them to get one value. We then examined the changes in gene expression level between the averaged value of carcinoma and normal, to get the most prominent changes. Figure 4 shows heatmaps for all 31 mice, of all of the transcripts that showed at least a four-fold change in the “average analysis”. It can clearly be seen that each of the mice has a unique pattern of transcriptional changes between C/N.

In the “heterogeneity analysis”, for each annotation, we looked at the ratios between the expression value of each transcript in carcinoma and normal in each mouse separately. This analysis emphasizes the differences between the mice (see below).

We compared the annotations from the two analyses that were denoted as significant. There were pronounced, biologically relevant differences between the analyses (**Table 1** **and** **2**). At the gene level, there were several cancer-related genes that were identified in a majority of the mice according to the heterogeneity analysis, but were not identified by the “average analysis” (**Table 1** **and** **2**). For example AKT1, a major anti-apoptotic gene, was not identified in the average analysis. In the heterogeneity analysis, AKT1 was prominent: it was induced in 10 mice, out of the 14 mice in which the DAVID annotation “regulation of programmed cell death” was significant. On the other hand, TP53 was identified in the average analysis, but was only altered in 5 of 14 mice in which “regulation of programmed cell death” was significant (**Table 1**). Similarly, for the annotation “cell cycle”, 8 genes that were significantly increased between C/N in more than half of the mice were not identified by the average analysis. Three genes that were significant in the average analysis were significantly induced in less than half of the mice (**Table 2**). These data demonstrate that small effects in a large number of samples can be ignored by the average analysis, whereas extreme changes in a minority of samples can have an undue effect on the average analysis. The mouse-by-mouse analysis gives a more informative picture of the significant changes, although it is of course much more tedious than the average analysis.

**Table 1:** “Average analysis” and “heterogeneity analysis” of cell death regulation reveal different genes.

Gene name	Number of mice	Gene name	Number of mice
***Il1b***	14	Bid	4
***Casp3***	13	Casp12	4
***Apaf1***	13	Ripk1	3
***Xiap***	12	Akt2	3
***Casp6***	12	Ngf	3
***Cflar***	11	Bax	3
Akt1	10	Ikbkb	3
***Ppp3r1***	10	Pik3cg	2
***Ppp3cb***	10	Tradd	2
Bcl2l1	10	Fadd	2
***Prkacb***	9	Csf2rb2	2
Csf2rb	8	Prkar1a	2
Traf2	7	Casp9	1
Il1rap	7	Atm	1
Pik3cd	6	Birc2	1
Capn1	6	Birc3	1
***Il1a***	6	Irak4	1
Prkar2b	5	Capn2	1
***Trp53***	5	Bad	1
Akt3	5	Casp7	1
Casp8	4		

Analysis of gene lists (KEGG) from all 14 mice in which the DAVID annotation “regulation of programmed cell death” was significant. Highlighted genes were also significant according to the average analysis of all 31 mice.

**Table 2:** “Average analysis” and “heterogeneity analysis” of cell cycle reveal different genes.

Gene name	Number of mice	Gene name	Number of mice
***Ccnb1***	16	Cdkn1a	5
***Ywhag***	16	Wee1	5
***Ccnd1***	16	Mcm6	4
***Tgfb1***	16	Smc1a	4
***Gm5593***	16	Mcm4	4
***Cdk1***	16	Mad1l1	4
***Bub1***	16	Cdc25b	4
***Cdc6***	15	Ywhaz	4
***Mcm2***	14	Cdc45	3
***Ccna2***	13	Cdc25a	3
Stag1	13	Sfn	3
***Mad2l1***	13	***Trp53***	3
***Cdk4***	13	Smad3	3
Mcm5	13	Chek2	3
Plk1	12	E2f1	2
Bub1b	12	Ywhah	2
***Cdkn2a***	12	Cdc23	2
***Ccnb2***	12	Pttg1	2
Anapc10	11	Ccne2	2
Cdc25c	10	Gadd45a	2
Cdc20	10	Rbl2	1
Rad21	10	Mdm2	1
Mcm7	8	Cdkn1c	1
Rbl1	8	Fzr1	1
***Bub3***	7	Atm	1
Ywhaq	7	Anapc1	1
Ccne1	7	Espl1	1
Skp2	7	Gadd45b	1
***Tgfb3***	7	Tfdp1	1
Ywhab	6	Anapc4	1
Chek1	6	Pkmyt1	1
Mcm3	6	Cdc26	1
Ttk	6	Cdc16	1
Ccnd2	6	Cdc27	1
Dbf4	5	Cdkn2d	1
Cdk6	5		

Analysis of gene lists (KEGG) from all 16 mice in which the DAVID annotation “cell cycle” was significant. Highlighted genes were also significant according to the average analysis of all 31 mice.

### Heterogeneity in cancer hallmarks: Comparison of two mice

In order to examine the role of heterogeneity in tumor progression in the individual mice, we looked at the specific transcripts that were significantly up-regulated or down-regulated between carcinoma and normal skin. For this purpose, we inserted a list of the significant genes for each mouse into the KEGG database. Only 49 genes were up-regulated and 37 genes were down-regulated in carcinoma vs. normal in all 31 mice. In each KEGG pathway, there were genes that were common to most or all of the mice, but most of the genes were significant in a minority of the mice.

To assess the role of heterogeneity in cancer, we examined the contribution to the cancer phenotype of the genes that were regulated in all the mice and of the genes that were regulated in only one or a few mice. To this end, we analyzed in depth the microarray data from two mice: Mouse ID7 and Mouse ID12. These mice were moderately, but not extremely, distant from one another in the heatmap shown in Figure 4. Both mice had developed papillomas by 10 weeks following treatment. The carcinomas from both mice were well differentiated, although mouse 7 had a class 1 tumor and mouse 12 had a class 2 tumor [see: GEO (GSE21264)]. The transcription of 417 genes was significantly enhanced, and the transcription of 375 genes significantly reduced, in the carcinomas from both mice relative to normal tail skin. The induced genes that were common to both mice included many genes that are important in the context of cancer (**Table 3** **and Table S2**). Yet, many more genes were induced in only one of the mice than were induced in both. 727 genes were up-regulated in Mouse ID7 but not in Mouse ID12, and 523 genes were up-regulated in Mouse ID12 but not in Mouse ID7 (**Table 3** **and Table S2**). 361 genes were significantly reduced between carcinoma and normal skin in Mouse ID7 but not in Mouse ID12, and 224 genes were reduced in Mouse ID12 but not in Mouse ID7. Like the common genes, many of the “mouse-specific” genes have a known involvement in cancer.

**Table 3:** Heterogeneity in cancer hallmarks - Comparison of Mouse ID7 and Mouse ID12.

Hallmark	Mouse ID 7	Mouse ID 12	Common
Sustaining Proliferative Signaling	FGF7, FGFR1, HGF, IGF2R, PDGFRA, PDGFRB	PGF, VEGFA, CCNB1, CCNE1, CDC25A, CDC6	IGF2BP2, HBEGF, CCNA2, CDK1
Evading Growth Suppressors		TGFBR1	TGFB1, TGFBR2
Resisting Cell Death	BCL11A, BCL2L11, AKT3, BCL2A1	XIAP, BCL2L15, MCL1	BCL3, IKBKE,
Inducing Angiogenesis	FGF7, PDGFRA, PDGFRB, CCL2, NRP1	VEGFA, TGFBR1	TGFB1, TGFBR2, TNFAIP2
Activating Invasion and Metastasis	CDH2, FOXC2, GNG11, MSN, SNAI1, VCAN, VPS13A,	SNAI3, SPARC,	AHNAK, BMP1, CALD1, COL1A2, CLO5A2, FN1, ITGA5, MMP3, MMP9, SERPINE1, STEAP1, WNT5A
Genome Instability and Mutation	BUB1B, BUB3	BUB1	
Tumor-Promoting Inflammation	TLR4, TRAF1,TRAF2, IFNAR2	IL1A, IL1RAP, TNFRSF12A, TNFSF9	IL1B, IL18RAP, IL6, TNFAIP2, TGFB1, SPP1, CXCL1, CXCL16, CXCL2, CXCL3
Reprogramming Energy Metabolism	ENO1, ENO3, PGAM2	PFKL	HK3
Evading Immune Destruction	IL10, PTGS1	VEGFA	TGFB1, PTGS2

The table displays central cancer hallmark genes [20] for which the expression level increased at least four-fold in one of the mice (Mouse ID7 or Mouse ID12) or in both mice.

We asked how the genes that were significantly altered in carcinoma in these two mice were related to the squamous cell carcinomas that the mice developed. Hanahan and Weinberg [20], [21] have defined several “hallmarks of cancer”. We therefore looked at genes involved in these hallmarks, and assessed genes with altered transcription in both mice and genes that were altered in only one of the mice (**Table 3**).

Sustained proliferative signaling is a central hallmark of cancer. Several central growth factors and cell cycle genes were transcriptionally induced in the carcinomas of both Mouse ID7 and Mouse ID12 (**Table 3**). With respect to the mouse-specific genes, in Mouse ID7 the growth factors PDGFRα, PDGFRβ, IGF2R and PDGFC were induced, whereas in mouse ID12 the growth factors TGFBR, PGF and VEGFA were induced. Further, in mouse ID12 cell cycle promoting genes, including CYCLIN B1, CYCLIN E1, CDC6 and CDC25a, were induced. Thus, in the carcinomas from both mice there is evidence for induction of sustained proliferative signaling, engendered by both shared and mouse-specific factors.

A related concept to sustained proliferation is the hallmark of enabling replicative immortality. There was no evidence for altered transcription of genes involved in telomere maintenance in either of the mice. Telomere maintenance may be affected by epigenetic mechanisms, which cannot be detected in expression microarrays.

Another hallmark of cancer is resisting cell death. Several anti-apoptotic genes were induced in both Mouse ID7 and Mouse ID12. In addition, several anti-apoptotic genes were induced in either Mouse ID7 or Mouse ID12. In mouse ID7, there was decreased transcription of phosphatidylinositol 3 kinase C (PIK3C), but there was a compensatory increase in transcription of AKT3. Thus, although some of the pathways were different, overall, there was an apparent increase in anti-apoptotic function in the carcinomas of both mice.

The transcription of several key inflammatory factors, including IL1β, IL6 and TGFβ1, was induced in the carcinomas of both mice. Nonetheless, extensive heterogeneity was observed between the two mice in other genes connected with inflammation (**Table** S**3**). In the carcinoma from Mouse ID7, there was strong induction at the level of transcription of numerous cytokines, chemokines and related genes. Mouse ID12 had induction of fewer genes, but these included major pro-inflammatory genes, such as IL1a and members of the TNF and TNFR families. A related hallmark, which also involves cytokines, is evasion of immune destruction of the tumor. IL10 is overexpressed in Mouse ID7, whereas VEGFA is overexpressed in Mouse ID12. Both of these can lead to immune suppression.

In addition to their roles in tumor-promoting inflammation, the pro-inflammatory genes also activate invasion and metastasis. Many additional genes that are characterized as promoting invasion and metastasis were transcriptionally induced in the carcinomas from both or either of the mice. FN1, BMP1, COL1A2, COL5A2, MMP3 and MMP9 were induced in tumors from both mice. Presumably, the fibronectins and collagens were induced in the tumor stroma. Thus, the tumors from both mice showed evidence of shared and mouse-specific transcriptional induction of genes promoting inflammation, invasiveness and metastasis.

The TGFβ pathway appears to be transcriptionally enhanced in both Mouse ID7 and ID12, which would be expected to lead to angiogenesis, another hallmark of cancer. It is notable that Mouse ID7 had increased PDGFR, whereas Mouse ID12 had an increase in VEGFR. These parallel pathways would both encourage angiogenesis. Overexpression of TGFβ could also lead to EMT and changes in cell-cell contacts, which could cause another hallmark, namely, evasion of growth suppression [22].

An important hallmark of cancer is reprogramming energy metabolism. Many metabolic genes were induced or repressed in both mice, and in each mouse separately (**Table S4**). As an example of how the cancers in Mouse ID7 and ID12 could evolve due to both shared and specific changes in metabolism, we looked at enzymes involved in glycolysis. Hexokinase 3 (HK3) was induced in both mice. Enolase (ENO1 and ENO3) and phosphoglycerate mutase 2 (PGAM2) were induced only in Mouse ID7, whereas phosphofructokinase (PFKL) was induced only in Mouse ID12. There was a decrease in transcription of several members of the Cytochrome P450 family in both mice, which was even more striking in Mouse ID7. This decrease would be expected to result in a decrease in aerobic respiration (**Table S5**).

In the article by Quigley et al. [23], the authors analyzed copy number changes in the tumors and showed evidence of genome instability, another hallmark of cancer. In our analysis of gene transcription, we found an increase in transcription of BUB1B and BUB3 in Mouse ID7, and a parallel increase in BUB1 transcription in Mouse ID12. Thus, the two mice had similar but different changes in transcription that could lead to genome instability. Although both p53 and pRB are considered to be essential “guardians of the genome”, they are frequently overexpressed in human tumors, and overexpression of p53 or pRB is often associated with poor prognosis [24], [25]. We note that Mouse ID7 had increased transcription of p53, whereas Mouse ID12 had increased transcription of pRBL1.

To summarize, the tumors from both Mouse ID7 and Mouse ID12 showed changes that were consistent with the hallmarks of cancer. Some of the transcriptional changes were shared, but many were specific to the individual mice.

## Discussion

The process of tumorigenesis is often highly heterogeneous, and a similar phenotype may arise from different molecular aberrations. Several recent studies have analyzed intra-tumor and inter-tumor heterogeneity in vivo and in vitro [26]–[30]. Accordingly, it should be taken into account that changes in gene expression during tumor development are inherently highly variable. When analyzing microarray data using the standard approaches, effects that are unique to only one or few of the animals may be overlooked. Contrarily, an exceptionally significant change in a single sample may shift the average and thus results may be misleading. Here, we examined the heterogeneity between different tumors, in an attempt to understand how different transcriptional alterations can lead to the same cancer phenotype. In each developing tumor there are many signals that can lead to many outcomes, and the most dominant ones will determine the tumor's fate.

In looking at the overall transcriptional patterns in the tumor, we are looking at a snapshot of the transcriptional activity at a given time (normal tail, papilloma or carcinoma). Although it is believed that tumor development is driven by a small number of “driver” mutations, thousands of “passenger” mutations are found by the time a tumor is detected. In the microarray analysis, we looked at overall changes in cellular activity, whether these were caused by drivers or by passengers. We saw extensive differences between all 31 mice, both in overall transcriptional changes and in the cellular functions that were significantly changed in each mouse.

**Figure 5:**
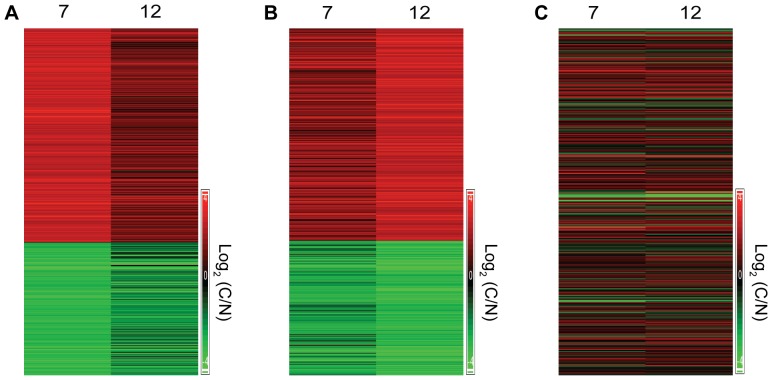
Heatmap of Mouse ID7 and Mouse ID12. A. Heatmap showing all transcripts that showed ≥4-fold change between carcinoma and normal skin in Mouse ID7 and their corresponding fold-change in Mouse ID12. B. Heatmap showing all transcripts that showed ≥4-fold change between carcinoma and normal skin in Mouse ID12 and their corresponding fold-change in Mouse ID12. C. Heatmap showing the fold-change of the transcripts that were significant in the average analysis, in Mouse ID7 and Mouse ID12.

Mouse ID7 and Mouse ID12 showed very different overall patterns of transcriptional change between carcinoma and normal skin (**Figure 5**). Yet, the detailed analysis of Mouse ID7 and Mouse ID12 provided a rational explanation for their shared cancer phenotype. When we analyzed the hallmarks of cancer [20], [21] we found that these were enhanced in both mice, both via shared pathways and via pathways that were unique to each mouse.

These findings are all the more surprising when one considers that the mice analyzed were extremely closely related to one another, and that the cancers were all caused by an identical treatment regime. In humans, of course, neither of these conditions pertains. Therefore, the degree of heterogeneity between human tumors is expected to be much greater. Our data emphasize the need for individualized cancer therapy. Individualized therapy requires appropriate tumor characterization, and the ability to choose tailored treatment that is appropriate to the molecular aberrations in the specific tumor.

To summarize, we found that mice sharing very similar genetic background, living in the same environment and treated with the same carcinogens develop the same kind of cancer, squamous cell carcinoma, but via different molecular mechanisms. We show that in each mouse different genes can participate in the hallmarks that lead to the tumorigenic phenotype, such that each tumor has a unique pattern of gene expression.

## Supporting Information

Table S1 **A:** DAVID annotations that were increased between normal skin and carcinoma (C/N) according to “heterogeneity analysis.” **B:** DAVID annotations that were decreased between normal skin and carcinoma (C/N) according to “heterogeneity analysis.”
(DOCX)

Table S2 Genes increased in at least 4-fold change between normal skin and carcinoma in Mouse ID7 and Mouse ID12.
(DOCX)

Table S3 Genes that were increased in at least 4-fold change and were involved in “Cytokine-cytokine receptor interaction” according to KEGG in mouse ID7 and mouse ID12.
(DOCX)

Table S4 Genes that were increased in at least 4-fold change and were involved in “Metabolic pathways” according to KEGG in mouse ID7 and mouse ID12.
(DOCX)

Table S5 Genes that were decreased in at least 4-fold change and were involved in “Metabolic pathways” according to KEGG in mouse ID7 and mouse ID12.
(DOCX)
