# Supplementary material for: Heterogeneity of Gene Expression in Murine Squamous Cell Carcinoma Development—The Same Tumor by Different Means
Source: PLoS One. 2013 Mar 18;8(3):e57748. doi: 10.1371/journal.pone.0057748 (PMC3601100; doi:10.1371/journal.pone.0057748)
Supplement: Table S1 — A: DAVID annotations that were increased between normal skin and carcinoma (C/N) according to “heterogeneity analysis.” B: DAVID annotations that were decreased between normal skin and carcinoma (C/N) according to “heterogeneity analysis.” (DOCX) [file pone.0057748.s001.docx]

**Table S1A**

| **Annotation** | **Number of mice** |
| --- | --- |
| cell migration | 31 |
| cell motion | 31 |
| cell motility | 31 |
| localization of cell | 31 |
| cell adhesion | 30 |
| angiogenesis | 30 |
| cell proliferation | 30 |
| gland development | 30 |
| tube morphogenesis | 30 |
| biological adhesion | 30 |
| vasculature development | 29 |
| cell chemotaxis | 29 |
| positive regulation of developmental process | 29 |
| leukocyte chemotaxis | 29 |
| response to wounding | 29 |
| tube development | 28 |
| blood vessel morphogenesis | 28 |
| morphogenesis of a branching structure | 28 |
| immune response | 28 |
| regulation of cell proliferation | 28 |
| inflammatory response | 27 |
| neutrophil chemotaxis | 27 |
| taxis | 27 |
| chemotaxis | 27 |
| blood vessel development | 27 |
| leukocyte migration | 25 |
| multicellular organismal metabolic process | 25 |
| epithelium development | 24 |
| multicellular organismal macromolecule | 24 |
| defense response | 24 |
| death | 23 |
| positive regulation of cell differentiation | 23 |
| cell death | 23 |
| collagen metabolic process | 23 |
| positive regulation of cellular | 23 |
| wound healing | 22 |
| apoptosis | 22 |
| actin cytoskeleton organization | 22 |
| programmed cell death | 21 |
| tissue morphogenesis | 21 |
| collagen catabolic process | 20 |
| positive regulation of cell motion | 20 |
| integrin-mediated signaling pathway | 20 |
| positive regulation of signal transduction | 20 |
| mammary gland development | 20 |
| multicellular organismal catabolic process | 19 |
| positive regulation of cell migration | 19 |
| positive regulation of cell proliferation | 19 |
| actin filament-based process | 19 |
| regulation of lymphocyte activation | 19 |
| protein amino acid phosphorylation | 19 |
| regulation of apoptosis | 19 |
| cytoskeleton organization | 19 |
| regulation of programmed cell death | 18 |
| positive regulation of locomotion | 18 |
| cell division | 18 |
| branching morphogenesis of a tube | 18 |
| regulation of cell death | 18 |
| regulation of cell migration | 18 |
| mitotic cell cycle | 18 |
| gland morphogenesis | 17 |
| embryonic development ending | 17 |
| cell activation | 17 |
| M phase of mitotic cell cycle | 17 |
| positive regulation of cell activation | 17 |
| skeletal system development | 17 |
| regulation of cell activation | 17 |
| organelle fission | 17 |
| regulation of leukocyte activation | 17 |
| nuclear division | 17 |
| cell cycle process | 17 |
| growth | 17 |
| mitosis | 17 |
| regulation of cell motion | 16 |
| regulation of cell adhesion | 16 |
| chordate embryonic development | 16 |
| leukocyte activation | 16 |
| extracellular matrix organization | 16 |
| M phase | 16 |
| cell cycle phase | 16 |
| positive regulation of cell communication | 16 |
| locomotory behavior | 16 |
| cell cycle | 16 |
| hemopoietic or lymphoid organ development | 16 |
| immune system development | 16 |
| positive regulation of immune system process | 15 |
| positive regulation of leukocyte activation | 15 |
| morphogenesis of an epithelium | 15 |
| positive regulation of lymphocyte activation | 15 |
| membrane invagination | 15 |
| endocytosis | 15 |
| hemopoiesis | 14 |
| extracellular structure organization | 14 |
| maintenance of protein location | 14 |
| in utero embryonic development | 14 |
| epidermal cell differentiation | 13 |
| membrane organization | 13 |
| maintenance of location | 13 |
| keratinocyte differentiation | 13 |
| epithelial cell differentiation | 13 |
| collagen fibril organization | 13 |
| cell-substrate adhesion | 13 |
| cell-matrix adhesion | 12 |
| myeloid leukocyte activation | 12 |
| regulation of organelle organization | 12 |
| regulation of phacytosis | 12 |
| immune effector process | 11 |
| adaptive immune response | 11 |
| negative regulation of cell communication | 11 |
| regulation of cellular component size | 11 |
| keratinization | 11 |
| positive regulation of T cell differentiation | 11 |
| positive regulation of apoptosis | 11 |
| adaptive immune response based on somatic | 11 |
| regulation of locomotion | 11 |
| heart development | 11 |
| chromosome segregation | 11 |
| enzyme linked receptor protein signaling pathway | 11 |
| regulation of T cell activation | 11 |
| regulation of adaptive immune response based on s | 10 |
| positive regulation of lymphocyte differentiation | 10 |
| innate immune response | 10 |
| negative regulation of signal transduction | 10 |
| regulation of phosphorylation | 10 |
| leukocyte mediated immunity | 10 |
| B cell mediated immunity | 10 |
| response to fungus | 10 |
| positive regulation of cell death | 10 |
| regulation of adaptive immune response | 10 |
| actin filament organization | 10 |
| T cell proliferation | 10 |
| regulation of cell cycle | 10 |
| positive regulation of programmed cell death | 10 |
| cell-cell adhesion | 10 |
| regulation of cytoskeleton organization | 9 |
| regulation of T cell differentiation | 9 |
| immunoglobulin mediated | 9 |
| ectoderm development | 9 |
| patterning of blood vessels | 9 |
| leukocyte homeostasis | 9 |
| vesicle-mediated transport | 9 |
| lymphocyte activation | 8 |
| leukocyte proliferation | 8 |
| regulation of kinase activity | 8 |
| behavior | 8 |
| lymphocyte mediated immunity | 8 |
| T cell activation | 8 |
| leukocyte adhesion | 8 |
| epidermis development | 8 |
| negative regulation of cell proliferation | 8 |
| positive regulation of phacytosis | 8 |
| regulation of lymphocyte differentiation | 8 |
| regulation of angiogenesis | 8 |
| mononuclear cell proliferation | 8 |
| phacytosis | 8 |
| lymphocyte homeostasis | 8 |
| phosphorus metabolic process | 7 |
| cytokine-mediated signaling pathway | 7 |
| positive regulation of cell adhesion | 7 |
| regulation of B cell activation | 7 |
| phosphate metabolic process | 7 |
| regulation of transferase activity | 7 |
| homeostasis of number of cells | 7 |
| positive regulation of endocytosis | 7 |
| antigen processing and presentation | 7 |
| positive regulation of T cell activation | 7 |
| regulation of protein kinase activity | 7 |
| positive regulation of response to stimulus | 7 |
| positive regulation of immune response | 7 |
| regulation of leukocyte mediated immunity | 7 |
| response to bacterium | 7 |
| cortical cytoskeleton organization | 7 |
| DNA metabolic process | 7 |
| antigen processing and presentation of | 7 |
| positive regulation of alpha-beta T cell activation | 6 |
| antigen processing and presentation of e | 6 |
| negative regulation of programmed cell death | 6 |
| negative regulation of cell death | 6 |
| positive regulation of cell-substrate adhesion | 6 |
| regulation of endocytosis | 6 |
| negative regulation of apoptosis | 6 |
| regulation of phosphate metabolic process | 6 |
| vascular endothelial growth factor receptor | 6 |
| myeloid cell differentiation | 6 |
| positive regulation of protein kinase cascade | 6 |
| regulation of immune effector process | 6 |
| regulation of lymphocyte mediated immunity | 6 |
| mast cell activation | 6 |
| phosphorylation | 6 |
| regulation of phosphorus metabolic process | 6 |
| glycoprotein biosynthetic process | 6 |
| cell cycle checkpoint | 6 |
| regulation of cell-substrate adhesion | 5 |
| tissue remodeling | 5 |
| embryonic placenta development | 5 |
| transmembrane receptor protein | 5 |
| negative regulation of cell differentiation | 5 |
| developmental growth | 5 |
| regulation of cell size | 5 |
| acute inflammatory response | 5 |
| response to estrogen stimulus | 5 |
| leukocyte differentiation | 5 |
| embryonic skeletal system morphogenesis | 5 |
| reproductive developmental process | 5 |
| glycoprotein metabolic process | 5 |
| cytokinesis | 5 |
| response to DNA damage stimulus | 5 |
| regulation of protein kinase cascade | 5 |
| placenta development | 5 |
| cellular response to stress | 5 |
| microtubule-based process | 5 |
| positive regulation of molecular function | 5 |
| epithelial tube morphogenesis | 5 |
| induction of apoptosis by intracellular signals | 5 |
| positive regulation of kinase activity | 5 |
| respiratory tube development | 5 |
| phacytosis; engulfment | 5 |
| muscle organ development | 4 |
| sister chromatid segregation | 4 |
| antigen processing and presentation | 4 |
| antigen processing and presentation of | 4 |
| intracellular signaling cascade | 4 |
| regulation of epithelial cell proliferation | 4 |
| regulation of B cell proliferation | 4 |
| interphase of mitotic cell cycle | 4 |
| positive regulation of alpha-beta | 4 |
| transforming growth factor beta receptor | 4 |
| mitotic sister chromatid segregation | 4 |
| muscle tissue development | 4 |
| regulation of morphogenesis of a branching structure | 4 |
| interphase | 4 |
| cell morphogenesis | 4 |
| regulation of response to external stimulus | 4 |
| positive regulation of organelle organization | 4 |
| mesenchymal-epithelial cell signaling | 4 |
| cartilage development | 4 |
| antigen processing and presentation of | 4 |
| cell differentiation involved in embryonic | 4 |
| regulation of cell cycle process | 4 |
| antigen processing and presentation of peptide | 4 |
| proteolysis | 4 |
| positive regulation of immune effector process | 4 |
| ossification | 4 |
| B cell homeostasis | 4 |
| DNA damage response; | 4 |
| positive regulation of adaptive | 3 |
| regulation of transforming | 3 |
| positive regulation of protein kinase activity | 3 |
| germ cell migration | 3 |
| regulation of leukocyte proliferation | 3 |
| positive regulation of B cell activation | 3 |
| induction of apoptosis | 3 |
| regulation of alpha-beta T cell activation | 3 |
| response to UV | 3 |
| respiratory system development | 3 |
| positive regulation of adaptive immune | 3 |
| regulation of nitric oxide biosynthetic process | 3 |
| regulation of hypersensitivity | 3 |
| positive regulation of transferase activity | 3 |
| regulation of mitotic cell cycle | 3 |
| embryonic organ development | 3 |
| regulation of B cell mediated immunity | 3 |
| acute-phase response | 3 |
| response to steroid hormone stimulus | 3 |
| regulation of immunoglobulin | 3 |
| activation of immune response | 3 |
| regulation of cytokine production | 3 |
| skeletal system morphogenesis | 3 |
| positive regulation of nitric oxide | 3 |
| DNA damage response; signal transduction | 3 |
| biomineral formation | 3 |
| cellular component morphogenesis | 3 |
| embryonic skeletal system development | 3 |
| regulation of acute inflammatory response | 3 |
| maintenance of location in cell | 3 |
| induction of programmed cell death | 3 |
| regulation of acute inflammatory response | 3 |
| cellular extravasation | 3 |
| negative regulation of cellular | 3 |
| lung development | 3 |
| regulation of actin cytoskeleton organization | 3 |
| transmembrane receptor protein | 3 |
| regulation of actin filament-based process | 3 |
| release of cytochrome c from mitochondria | 3 |
| regulation of alpha-beta T cell differentiation | 3 |
| response to endoplasmic reticulum stress | 3 |
| urogenital system development | 3 |
| response to molecule of bacterial origin | 3 |
| DNA replication initiation | 2 |
| regulation of type II hypersensitivity | 2 |
| macrophage activation | 2 |
| face development | 2 |
| DNA repair | 2 |
| negative regulation of transforming growth | 2 |
| kidney development | 2 |
| epithelial cell proliferation | 2 |
| regulation of lymphocyte proliferation | 2 |
| B cell receptor signaling pathway | 2 |
| DNA recombination | 2 |
| mammary gland duct morphogenesis | 2 |
| bone development | 2 |
| regulation of tumor necrosis factor production | 2 |
| T cell homeostasis | 2 |
| positive regulation of type IIa hypersensitivity | 2 |
| regulation of mononuclear cell proliferation | 2 |
| regulation of MAP kinase activity | 2 |
| positive regulation of myeloid | 2 |
| regulation of inflammatory response | 2 |
| striated muscle tissue development | 2 |
| organelle localization | 2 |
| establishment of organelle localization | 2 |
| antigen processing and presentation of | 2 |
| regulation of cell morphogenesis | 2 |
| lymphocyte proliferation | 2 |
| regulation of CD4-positive | 2 |
| response to ionizing radiation | 2 |
| cell morphogenesis involved in differentiation | 2 |
| negative regulation of cell growth | 2 |
| positive regulation of CD4-positive; | 2 |
| positive regulation of type II hypersensitivity | 2 |
| reproductive structure development | 2 |
| regulation of mesenchymal cell proliferation | 2 |
| microtubule cytoskeleton organization | 2 |
| regulation of type IIa hypersensitivity | 2 |
| positive regulation of angiogenesis | 2 |
| cell projection organization | 2 |
| maintenance of protein location in cell | 2 |
| response to hypoxia | 2 |
| unsaturated fatty acid metabolic process | 2 |
| lymphocyte differentiation | 2 |
| DNA integrity checkpoint | 2 |
| mitotic cell cycle checkpoint | 2 |
| mesenchyme development | 2 |
| axon guidance | 2 |
| receptor-mediated endocytosis | 2 |
| negative regulation of angiogenesis | 2 |
| positive regulation of cytokine production | 2 |
| response to organic substance | 2 |
| regulation of vesicle-mediated transport | 2 |
| mammary gland morphogenesis | 2 |
| spindle organization | 1 |
| negative regulation of protein kinase activity | 1 |
| negative regulation of transport | 1 |
| cellular protein complex assembly | 1 |
| mesenchymal cell differentiation | 1 |
| positive regulation of mesenchymal cell proliferation | 1 |
| apoptotic cell clearance | 1 |
| keratinocyte proliferation | 1 |
| positive regulation of immunoglobulin | 1 |
| ER-nuclear signaling pathway | 1 |
| epidermal growth factor receptor signaling pathway | 1 |
| regulation of T cell proliferation | 1 |
| protein processing | 1 |
| embryonic morphogenesis | 1 |
| negative regulation of transferase activity | 1 |
| negative regulation of immune system process | 1 |
| cell recognition | 1 |
| regulation of actin filament length | 1 |
| branching involved in mammary | 1 |
| cellular macromolecular complex assembly | 1 |
| negative regulation of DNA metabolic process | 1 |
| regulation of microtubule-based process | 1 |
| chondrocyte differentiation | 1 |
| chondroitin sulfate proteoglycan metabolic process | 1 |
| negative regulation of response to stimulus | 1 |
| RNA processing | 1 |
| positive regulation of leukocyte proliferation | 1 |
| cellular response to unfolded protein | 1 |
| cellular macromolecular complex subunit organization | 1 |
| regulation of I-kappaB kinase/NF-kappaB cascade | 1 |
| regulation of MAPKKK cascade | 1 |
| embryonic hemopoiesis | 1 |
| negative regulation of macromolecule metabolic process | 1 |
| negative regulation of growth | 1 |
| muscle contraction | 1 |
| aminoglycan metabolic process | 1 |
| exocrine system development | 1 |
| endoplasmic reticulum unfolded protein response | 1 |
| protein maturation by peptide bond cleavage | 1 |
| polysaccharide metabolic process | 1 |
| negative regulation of leukocyte activation | 1 |
| defense response to bacterium | 1 |
| DNA-dependent DNA replication | 1 |
| regulation of microtubule cytoskeleton organization | 1 |
| response to hormone stimulus | 1 |
| negative regulation of kinase activity | 1 |
| protein kinase cascade | 1 |
| mesenchymal cell development | 1 |
| chondroitin sulfate metabolic process | 1 |
| mRNA processing | 1 |
| protein maturation | 1 |
| positive regulation of leukocyte mediated immunity | 1 |
| neuron projection development | 1 |
| regulation of body fluid levels | 1 |
| positive regulation of B cell proliferation | 1 |
| icosanoid metabolic process | 1 |
| sphinlipid metabolic process | 1 |
| positive regulation of transport | 1 |
| nuclear transport | 1 |
| salivary gland morphogenesis | 1 |
| lipoprotein metabolic process | 1 |
| regulation of DNA metabolic process | 1 |
| neural crest cell development | 1 |
| iron ion transport | 1 |
| regulation of establishment o | 1 |
| positive regulation of lymphocy | 1 |
| hemostasis | 1 |
| regulation of cell shape | 1 |
| response to oxygen levels | 1 |
| double-strand break repair | 1 |
| positive regulation of multicellular | 1 |
| positive regulation of defense response | 1 |
| muscle system process | 1 |
| immune response-regulating cell surface | 1 |
| response to mechanical stimulus | 1 |
| regulation of homeostatic process | 1 |
| negative regulation of molecular function | 1 |
| neural crest cell differentiation | 1 |
| nucleocytoplasmic transport | 1 |
| positive regulation of cell cycle | 1 |
| positive regulation of B cell | 1 |
| antigen processing and presentation | 1 |
| DNA replication | 1 |
| negative regulation of cell size | 1 |
| pattern specification process | 1 |
| branching involved in salivary | 1 |
| immune response-regulating | 1 |
| macromolecular complex assembly | 1 |
| negative regulation of cell activation | 1 |
| regulation of T cell mediated cytotoxicity | 1 |
| response to endogenous stimulus | 1 |
| blood coagulation | 1 |
| coagulation | 1 |
| regulation of protein localization | 1 |
| chromosome organization | 1 |
| regulation of myeloid leukocyte | 1 |
| blastocyst development | 1 |
| positive regulation of mononuclear | 1 |
| anti-apoptosis | 1 |
| neuron development | 1 |
| embryonic cranial skeleton morphogenesis | 1 |
| regulation of actin polymerization | 1 |
| mRNA metabolic process | 1 |
| protein localization | 1 |
| negative regulation of lymphocyte activation | 1 |
| tissue regeneration | 1 |
| membrane lipid metabolic process | 1 |
| positive regulation of cellular | 1 |
| metaphase plate congression | 1 |
| regulation of inflammatory response | 1 |
| positive regulation of NF-kappaB | 1 |
| DNA damage checkpoint | 1 |
| female sex differentiation | 1 |
| mammary gland duct branch elongation | 1 |
| protein polymerization | 1 |
| negative regulation of epithelial | 1 |
| humoral immune response | 1 |
| DNA damage response; signal | 1 |
| regulation of epidermis development | 1 |
| positive regulation of biosynthetic process | 1 |
| positive regulation of lymphocyte | 1 |
| macromolecular complex subunit | 1 |
| regulation of branching involved in | 1 |
| regeneration | 1 |
| chromosome localization | 1 |
| establishment of chromosome localization | 1 |

**Table S1B**

| **Annotation** | **Number of mice** |
| --- | --- |
| fatty acid metabolic process | 31 |
| oxidation reduction | 31 |
| fatty acid biosynthetic process | 30 |
| lipid biosynthetic process | 30 |
| secondary metabolic process | 29 |
| organic acid biosynthetic process | 27 |
| carboxylic acid biosynthetic process | 27 |
| lipid catabolic process | 26 |
| epidermis development | 25 |
| brown fat cell differentiation | 25 |
| ectoderm development | 24 |
| retinoid metabolic process | 23 |
| terpenoid metabolic process | 23 |
| diterpenoid metabolic process | 23 |
| fat-soluble vitamin metabolic process | 21 |
| fat cell differentiation | 20 |
| isoprenoid metabolic process | 19 |
| molting cycle | 17 |
| hair cycle | 17 |
| vitamin A metabolic process | 16 |
| cellular hormone metabolic process | 16 |
| retinol metabolic process | 16 |
| vitamin metabolic process | 15 |
| steroid metabolic process | 14 |
| cellular lipid catabolic process | 14 |
| steroid biosynthetic process | 11 |
| phospholipid metabolic process | 11 |
| regulation of hormone levels | 10 |
| hormone metabolic process | 10 |
| organophosphate metabolic process | 10 |
| epithelium development | 9 |
| striated muscle tissue development | 8 |
| cholesterol metabolic process | 8 |
| epithelial cell differentiation | 8 |
| epidermal cell differentiation | 8 |
| keratinization | 8 |
| keratinocyte differentiation | 8 |
| muscle organ development | 7 |
| regionalization | 7 |
| membrane lipid metabolic process | 7 |
| proximal/distal pattern formation | 7 |
| muscle tissue development | 7 |
| sterol metabolic process | 7 |
| skeletal muscle tissue development | 6 |
| muscle system process | 6 |
| appendage development | 6 |
| limb morphogenesis | 6 |
| sterol biosynthetic process | 6 |
| skeletal muscle organ development | 6 |
| tissue morphogenesis | 6 |
| cholesterol biosynthetic process | 6 |
| melanin metabolic process | 6 |
| melanin biosynthetic process | 6 |
| limb development | 6 |
| appendage morphogenesis | 6 |
| isoprenoid biosynthetic process | 5 |
| sphingolipid metabolic process | 5 |
| skin development | 5 |
| negative regulation of cell proliferation | 5 |
| hair cycle process | 5 |
| hair follicle development | 5 |
| organic ether metabolic process | 5 |
| cell-cell adhesion | 5 |
| molting cycle process | 5 |
| skeletal system development | 5 |
| muscle contraction | 5 |
| fatty acid catabolic process | 4 |
| actomyosin structure organization | 4 |
| benzene and derivative metabolic process | 4 |
| urogenital system development | 4 |
| neutral lipid metabolic process | 4 |
| acute-phase response | 4 |
| acute inflammatory response | 3 |
| cartilage development | 3 |
| inflammatory response | 3 |
| cell adhesion | 3 |
| triglyceride metabolic process | 3 |
| biological adhesion | 3 |
| myofibril assembly | 3 |
| morphogenesis of an epithelium | 3 |
| embryonic limb morphogenesis | 3 |
| embryonic appendage morphogenesis | 3 |
| glycerol ether metabolic process | 3 |
| muscle cell differentiation | 3 |
| bone development | 3 |
| intermediate filament-based process | 3 |
| intermediate filament cytoskeleton organization | 3 |
| acylglycerol metabolic process | 3 |
| anterior/posterior pattern formation | 3 |
| retinoic acid metabolic process | 2 |
| carboxylic acid catabolic process | 2 |
| complement activation | 2 |
| pigment biosynthetic process | 2 |
| extracellular matrix organization | 2 |
| protein maturation by peptide bond cleavage | 2 |
| lipid localization | 2 |
| muscle cell development | 2 |
| regulation of body fluid levels | 2 |
| blood coagulation | 2 |
| striated muscle cell differentiation | 2 |
| proteoglycan metabolic process | 2 |
| wound healing | 2 |
| extracellular structure organization | 2 |
| pattern specification process | 2 |
| regulation of coagulation | 2 |
| pigment metabolic process | 2 |
| activation of plasma proteins involved in acute inflammatory response | 2 |
| complement activation; alternative pathway | 2 |
| regulation of blood coagulation | 2 |
| neuron differentiation | 2 |
| lipid transport | 2 |
| defense response | 2 |
| response to wounding | 2 |
| striated muscle cell development | 2 |
| aromatic amino acid family metabolic process | 2 |
| xenobiotic metabolic process | 2 |
| tyrosine metabolic process | 2 |
| humoral immune response | 2 |
| cellular component assembly involved in morphogenesis | 2 |
| hemostasis | 2 |
| organic acid catabolic process | 2 |
| purine nucleotide metabolic process | 2 |
| humoral immune response mediated by circulating immunoglobulin | 2 |
| white fat cell differentiation | 2 |
| coagulation | 2 |
| complement activation; classical pathway | 2 |
| transmission of nerve impulse | 1 |
| hexose biosynthetic process | 1 |
| regulation of response to external stimulus | 1 |
| cellular aldehyde metabolic process | 1 |
| sensory organ development | 1 |
| cholesterol transport | 1 |
| intermediate filament organization | 1 |
| calcium ion homeostasis | 1 |
| peptide cross-linking | 1 |
| glycerolipid metabolic process | 1 |
| establishment of planar polarity | 1 |
| protein maturation | 1 |
| innate immune response | 1 |
| response to hormone stimulus | 1 |
| homophilic cell adhesion | 1 |
| neuron development | 1 |
| regulation of cell proliferation | 1 |
| metal ion homeostasis | 1 |
| actin cytoskeleton organization | 1 |
| regulation of ossification | 1 |
| pyruvate metabolic process | 1 |
| sterol transport | 1 |
| enzyme linked receptor protein signaling pathway | 1 |
| response to peptide hormone stimulus | 1 |
| cardiac muscle tissue development | 1 |
| aromatic compound catabolic process | 1 |
| morphogenesis of a polarized epithelium | 1 |
| regulation of lipid metabolic process | 1 |
| monosaccharide biosynthetic process | 1 |
| amine catabolic process | 1 |
| ATP metabolic process | 1 |
| amine biosynthetic process | 1 |
| regulation of epithelial cell proliferation | 1 |
| protein processing | 1 |
| chondrocyte differentiation | 1 |
| neutral lipid catabolic process | 1 |
| gluconeogenesis | 1 |
| mammary gland development | 1 |
| ossification | 1 |
| immunoglobulin mediated immune response | 1 |
| response to endogenous stimulus | 1 |
| cellular metal ion homeostasis | 1 |
| positive regulation of cell differentiation | 1 |
| homeostatic process | 1 |
| response to steroid hormone stimulus | 1 |
| cellular amino acid catabolic process | 1 |
| negative regulation of blood coagulation | 1 |
