# Supplementary material for: Heterogeneity of Gene Expression in Murine Squamous Cell Carcinoma Development—The Same Tumor by Different Means
Source: PLoS One. 2013 Mar 18;8(3):e57748. doi: 10.1371/journal.pone.0057748 (PMC3601100; doi:10.1371/journal.pone.0057748)
Supplement: Table S2 — Genes increased in at least 4-fold change between normal skin and carcinoma in Mouse ID7 and Mouse ID12. (DOCX) [file pone.0057748.s002.docx]

**Table S2**

| **Genes increased only in mouse ID7** | **Genes increased only in mouse ID12** | **Genes increased in both mice** |
| --- | --- | --- |
| AA414768 | AA409587 | AB124611 |
| AA986860 | Acadl | Adam12 |
| Abcc9 | Acp5 | Adam8 |
| Abcd1 | Acsl4 | Adamts12 |
| Abcg1 | Acta1 | Adamts4 |
| Abra | Actn1 | Adcy7 |
| Ace | Adamts2 | Adora1 |
| Acot7 | Adarb1 | Adrbk2 |
| Acp2 | Add2 | Afg3l2 |
| Acsl3 | Adm | Ahnak |
| Actn2 | Ahcy | Aif1 |
| Actn3 | Aknad1 | Ak1 |
| Adal | Akr1b3 | Akr1b10 |
| Adam19 | Akr1b8 | Amigo1 |
| Adamts1 | Aldh1a3 | Anxa3 |
| Adamts5 | Aldh7a1 | Ap2a2 |
| Adamts6 | Anapc10 | Apaf1 |
| Adamts7 | Anxa1 | Aplnr |
| Adap2 | Apobec1 | Apob48r |
| Adcy9 | Apol7a | Apobec3 |
| Adora2a | Apold1 | Areg |
| Adssl1 | Appbp2 | Arhgef2 |
| Agmat | Aqp3 | Armcx3 |
| Agps | Aqp5 /// LOC100046616 | Arntl2 |
| Agtr2 | Arc | Aspm |
| Ahcyl2 | Arg1 | AU015263 |
| AI120166 | Armc9 | Auh |
| AI256396 | Arpc2 | B4galnt1 |
| AI447881 | Atcay | B4galt6 |
| AI504432 | Atf3 | BC055324 |
| AI593442 | Atf7 | Bcat1 |
| AI605517 | Atf7ip | Bcl3 |
| AI747699 | Atp13a3 | Bdkrb2 |
| Airn | Atp1b2 | Bhlhe22 |
| Akap2 | Atp5h | Bmp1 |
| Akt3 | AU022252 | Bst1 |
| Aldh18a1 | AU042651 | C1qtnf3 |
| Alox5ap | Aurka | C5ar1 |
| Amot | Aurkb | Cald1 |
| Angptl2 | Auts2 | Car4 |
| Ankrd1 | B3gnt3 | Casp3 |
| Ankrd2 | Bak1 | Cbx5 |
| Ankrd55 | Bcl2l15 | Ccbe1 |
| Ankrd6 | Birc5 | Ccdc102a |
| Anxa5 | Bst1 | Ccl12 |
| Anxa6 | Bub1 | Ccl3 |
| Ap1s2 | C1galt1 | Ccl8 |
| Ap3s2 | C1qtnf5 /// Mfrp | Ccl9 |
| Apobec2 | C79407 | Ccna2 |
| Aqp1 | Calcb | Ccnd1 |
| Aqp2 | Calm3 | Ccr1 |
| Arap3 | Cand1 | Ccr5 |
| Arfgap1 | Cap1 | Cd14 |
| Arhgap30 | Capn1 | Cd177 |
| Arhgap4 | Capn5 | Cd209e |
| Arhgdib | Car13 | Cd276 |
| Arl13b | Car5b | Cd300a |
| Armcx2 | Carhsp1 | Cd300lb |
| Art1 | Casc4 | Cd300ld |
| Asb15 | Ccdc33 | Cd40 |
| Asb5 | Ccdc46 | Cd52 |
| Asph | Ccdc50 | Cd53 |
| Athl1 | Ccdc88b | Cd83 |
| Atp10a | Ccdc97 | Cdk1 |
| Atp1a2 | Ccnb1 /// Gm5593 /// Gm8416 | Cenpe |
| Atp2a1 | Ccnb1ip1 | Cep55 |
| Atp6v0d2 | Ccne1 | Cfp |
| Atp8b4 | Ccng1 | Chi3l3 |
| AU042950 | Cd2bp2 | Chi3l3 /// Chi3l4 |
| AW555355 | Cd4 | Chst2 |
| AW987390 | Cd44 | Cks1b |
| B3galnt1 | Cd80 | Clcf1 |
| Bace1 | Cdadc1 | Clec4a2 |
| Bbx | Cdc25a | Clec4d |
| BC025446 | Cdc42bpa | Clec4e |
| Bcl11a | Cdc5l | Clec4n |
| Bcl2a1a /// Bcl2a1b | Cdc6 | Clec5a |
| Bcl2l11 | Cdca5 | Clec7a |
| Bdnf | Cdk18 | Cln3 |
| Bend6 | Cdkn2a | Cnn2 |
| Bgn | Cdv3 | Col1a1 |
| Bin1 | Ceacam1 | Col1a2 |
| Bmyc | Ceacam1 /// Ceacam2 | Col4a1 |
| Bnc2 | Ceacam10 | Col5a1 |
| Bola2 | Celf4 | Col5a2 |
| Bptf | Cenpn | Col5a3 |
| Btg1 | Cflar | Col6a2 |
| Btk | Cgref1 | Col6a3 |
| Bub1b | Chd6 | Col8a1 |
| Bub3 | Chi3l4 | Coro1a |
| C1qa | Chid1 | Cpxm1 |
| C1qb | Cisd3 | Crabp1 |
| C1qc | Ckap2l | Creb3l2 |
| C3 | Clcn5 | Csf3 |
| C3ar1 | Cldn4 | Csf3r |
| C6 | Cldn7 | Csrp2 |
| C81615 | Cldn8 | Cthrc1 |
| Calhm2 | Clstn2 | Ctla2a |
| Calm1 | Cntd1 | Ctnnbip1 |
| Calml4 | Col4a3bp | Cxcl1 |
| Calu | Col7a1 | Cxcl16 |
| Camk1 | Copg | Cxcl2 |
| Camk1g | Cotl1 | Cxcl3 |
| Camkk2 | Cox7a2l | Cxcr2 |
| Casp12 | Cpn1 | Cxcr2 |
| Casp4 | Crisp1 | Cxcr4 |
| Casq1 | Ctla2a /// Ctla2b | Cyp4f18 |
| Catsperg1 | Ctsc | Cyr61 |
| Cbx6 | Cux1 | Cyth4 |
| Ccdc126 | Cxcl9 | Cytsb |
| Ccdc88a | Cxxc1 | D0H4S114 |
| Ccdc9 | Cyb5b | Dclk1 |
| Ccl11 | Cyb5r3 | Ddah1 |
| Ccl2 | D2Ertd750e | Dhps |
| Ccl24 | Ddx21 | Dmrta2 |
| Ccl4 | Ddx39 | Dnajb4 |
| Ccl5 | Ddx6 | Dtx3l |
| Ccl6 | Degs2 | Dusp6 |
| Ccl7 | Depdc1a | Dusp9 |
| Cd163 | Depdc7 | Dzip3 |
| Cd200r1 | Dgka | E2f7 |
| Cd209c | Dhx16 | E2f8 |
| Cd209f | Diap3 | Eef1d |
| Cd209g | Dnajc18 | Efemp2 |
| Cd244 | Dnajc5 | Ehd2 |
| Cd247 | Dsc2 | Eif2ak2 |
| Cd33 | Dscc1 | Elk3 |
| Cd34 | Dtl | Enpp1 |
| Cd84 | Dusp5 | Epgn |
| Cd86 | Dusp7 | Eps8 |
| Cdh2 | Dyrk3 | Eps8 |
| Cdk20 | Ece1 | Epsti1 |
| Celf6 | Ecm1 | Ept1 |
| Cep170 | Ehd1 | Ercc1 |
| Ch25h | Eif2b2 | Etnk1 |
| Chrna1 | Eif3a | Ets1 |
| Chst7 | Eif4b | F10 |
| Cit | Eif4ebp1 | Fam111a |
| Ckm | Eif4enif1 | Fam120b |
| Clec1a | Eif4g1 | Fblim1 |
| Clip4 | Elf3 | Fbln2 |
| Cmah | Elf5 | Fcgr1 |
| Cml3 | Eny2 | Fcgr2b |
| Cmtm3 | Epha2 | Fcgr4 |
| Cnn3 | Erc1 | Fcrls |
| Cnnm2 | Ercc6l | Fgr |
| Col24a1 | Ergic1 | Flrt2 |
| Col4a2 | Eri2 | Fn1 |
| Col6a1 | Esd | Fos |
| Cox6a2 | Esm1 | Fosl1 |
| Cox7a1 | Exo1 | Foxg1 |
| Cox8b | F5 | Fpr1 |
| Cp | Fabp4 | Fpr2 |
| Cpa3 | Fam107a | Fscn1 |
| Cr1l | Fam120a | Fyb |
| Creb3l1 | Fam13b | Gap43 |
| Crem | Fam176a | Gcnt1 |
| Csf1r | Fam23a | Gcnt2 |
| Csf2ra | Fam49b | Gfpt1 |
| Csgalnact1 | Fam60a | Gins2 |
| Csrp3 | Fam73a | Glipr1 |
| Ctla2a /// Ctla2b | Fam83d | Lilrb3 /// Pira1 /// Pira11 /// Pira2 /// Pira4 |
| Ctla2b | Far1 | Gm10883 |
| Ctss | Fastkd2 | Gm9844 /// Tmsb10 |
| Ctsz | Fbn2 | Gpd2 |
| Cul3 | Fignl1 | Gpr65 |
| Cyba | Flnb | Gpr85 |
| Cybb | Fmo4 | Gsr |
| Cyp2c65 | Fosb | H19 |
| Cyp4f16 | Foxa1 | H2afv |
| Cyp7b1 | Foxp1 | Has2 |
| Cytip | Fst | Havcr2 |
| Dab2 | Fut10 | Hbegf |
| Dag1 | G0s2 | Hck |
| Dapk1 | G2e3 | Hcls1 |
| Dcps | Gadl1 | Hdc |
| Ddr2 | Galnt3 | Hk3 |
| Ddx58 | Galnt7 | Hmga2 |
| Ddx60 | Gdnf | Hmmr |
| Dennd1c | Ggcx | Hoxa1 |
| Dhx58 | Ggta1 | Hoxc5 |
| Dicer1 | Gigyf2 | Hp |
| Dio2 | Gkn1 | Htra1 |
| Dis3 | Glb1 | Htra2 |
| Dkk2 | Glyat | Ier5l |
| Dkk3 | Gm11276 /// Hist1h2ad /// Hist1h2ao | Ifi202b |
| Dmp1 | Serpinb9e /// Serpinb9f | Ifi203 |
| Dmrta1 | Gm5081 | Ifi204 |
| Dnajb13 | Gm6742 /// Odc1 | Ifitm1 |
| Dnm1 | Gm6948 /// Rps24 | Ifitm6 |
| Dnm3os | Gmfb | Igf2bp2 |
| Dock2 | Gmppb | Igh-2 /// Igh-VJ558 |
| Dock6 | Gnpda2 | Il18rap |
| Dock8 | Gp49a /// Lilrb4 | Il1b |
| Dok1 | Gpr64 | Il1rl1 |
| Dok2 | Gprc5a | Il2rg |
| Dpysl3 | Grb2 | Il4ra |
| Dusp3 | Gsdmcl2 | Il6 |
| Dysf | Gsta1 /// Gsta2 | Inhba |
| Ear1 /// Ear12 | Gsta4 | Ipcef1 |
| Ear11 | Gsto1 | Irf7 |
| Ear2 | Gzmd /// Gzme | Islr2 |
| Ear3 | Gzme | Itga1 |
| Ebf3 | Has3 | Itga5 |
| Ednra | Haus6 | Itgam |
| Efcab9 | Hdlbp | Itgax |
| Ehbp1l1 | Hells | Itgb1 |
| Elavl2 | Hivep2 | Itgb2 |
| Emilin1 | Hmga1 /// Hmga1-rs1 | Itgb2l |
| Emp3 | Hnf1b | Kcnn4 |
| Emr1 | Hnrnpc | Kcnq5 |
| Enc1 | Hpgds | Kdelr3 |
| Eno1 | Hs3st1 | Kif13b |
| Eno3 | Hs6st2 | Kif22 |
| Entpd1 | Hsp90aa1 | Kif2c |
| Epha3 | Hsph1 | Klhl17 |
| Epha4 | Hus1 | Krt18 |
| Ephb2 | Ifi203 /// Ifi204 /// Mnda /// Mndal | Krt8 |
| Etv1 | Ifi205 /// Mnda | Lamb1-1 |
| Evi2a | Ifnz | Lamc1 |
| Evl | Ikbke | Lasp1 |
| Ext1 | Ikzf2 | Lbh |
| F13a1 | Il1a | Lclat1 |
| F7 | Il1rap | Lcp1 |
| Fabp3 | Il23a | Leng8 |
| Fam114a1 | Incenp | Lepre1 |
| Fam13c | Insl6 | Lgals1 |
| Fam149a | Isg20 | Lgi2 |
| Fam167b | Itga2 | Lipg |
| Fam176b | Itga3 | Lmnb1 |
| Fam83h | Itga6 | Sirpb1a /// Sirpb1b |
| Fanci | Itgb6 | Lyn |
| Fbn1 | Kcne3 | Loxl2 |
| Fbxo24 | Kcnk1 | Loxl3 |
| Fbxo42 | Kdm5b | Lpcat1 |
| Fbxw2 | Kif11 | Lpgat1 |
| Fcer1g | Kif20a | Lpp |
| Fcgr3 | Kif20b | Lrrc47 |
| Fcgrt | Kif23 | Lrrc67 |
| Fcna | Kirrel | Malat1 |
| Fermt3 | Klc1 | Map3k10 |
| Fes | Klf7 | Map3k7 |
| Fez1 | Klk1 | Mapkap1 |
| Fgd2 | Klk10 | Marcksl1 |
| Fgf7 | Klk12 | Mcpt8 |
| Fgfr1 | Klk8 | Mefv |
| Fhl1 | Klra2 | Meg3 |
| Fkbp10 | Kpna2 | Melk |
| Fkbp11 | Kpna4 | Met |
| Fkrp | Krt13 | Mex3b |
| Flnc | Krt16 | Mgat3 |
| Flt1 | Krt19 | Mlec |
| Fmnl2 | Krt4 | Mmp10 |
| Fndc3b | Krt6b | Mmp12 |
| Folr2 | Krt7 | Mmp13 |
| Foxc2 | Lamb3 | Mmp14 |
| Fstl1 | Lamc2 | Mmp19 |
| Fth1 | Lamp3 /// Ppid | Mmp3 |
| Fut8 | Larp4 | Mmp8 |
| Galk1 | Lce3a | Mmp9 |
| Galnt10 | Lima1 | Mrgpra2a /// Mrgpra2b |
| Galnt2 | LOC100045487 /// Nkx6-1 | Ms4a4c |
| Ganc | LOC100045999 /// Ran | Ms4a4d |
| Gas5 | LOC100046891 /// Smad5 | Ms4a6d |
| Gbp2 | LOC100047199 /// Odf2 | Ms4a7 |
| Gbp3 | LOC100048538 /// Prss27 | Msr1 |
| Gcg | LOC630729 | Mtap |
| Gimap4 | Rpl13a | Myadm |
| Glcci1 | LOC676960 /// Prrg4 | Myef2 |
| Glipr2 | LOC676974 | Myo1b |
| Gm14085 | LOC677213 /// Uhmk1 | Myo1g |
| Gm2115 | LOC677213 /// Uhmk1 | Nans |
| Gm22 | LOC73899 | Ncf1 |
| Gm5571 /// Igk-V1 | Lpcat4 | Ncf2 |
| Gm7202 /// Igk-V19-14 | Lpin2 | Ncf4 |
| Gm9187 | Lrrc26 | Nfkbie |
| Gmfg | Lrrc28 | Ngp |
| Gnb4 | Ltf | Nox4 |
| Gng11 | Ly6e | Nqo2 |
| Gng2 | Ly6k | Nts |
| Gp49a /// Lilrb4 | Mad2l1 | Oaf |
| Gpc6 | Mal | Oasl2 |
| Gpr114 | Mall | Olfm4 |
| Gpr124 | Malt1 | Osgepl1 |
| Gpr149 | Mamstr | Osm |
| Gpr171 | Map4k4 | Parva |
| Gprc5b | Map4k5 | Pcdh7 |
| Gpsm3 | Mast4 | Pcsk5 |
| Grasp | Mat2a | Pdia4 |
| Grem1 | Mbnl3 | Pdpn |
| Gspt2 | Mboat1 | Pglyrp1 |
| Gtpbp3 | Mcf2l | Phlda1 |
| Gusb | Mcl1 | Pi15 |
| Gxylt2 | Mcm5 | Pilrb1 |
| Gzf1 | Mdfi | Pira2 |
| Gzma | Med8 | Pitpnc1 |
| Gzmb | Mettl4 | Pitx1 |
| H13 | Mfap4 | Pja1 |
| H2-Ea | Mgam | Pla1a |
| H2-Q7 | Mmgt2 | Plac8 |
| H60a | Morn4 | Plaur |
| Has1 | Mpeg1 | Plod2 |
| Herc5 | Mpzl2 | Plscr1 |
| Hexa | Mrps11 | Pmepa1 |
| Hfe | Mrps25 | Pml |
| Hfe2 | Mrrf | Prc1 |
| Hgf | Msln | Prkrir |
| Hic1 | Mthfd2 | Prok2 |
| Hipk2 | Mtm1 | Prokr2 |
| Hmcn1 | Mtpn | Prr11 |
| Hmga2-ps1 | Mxd1 | Prrx1 |
| Hmgcr | Myl7 | Psg28 |
| Hoxc4 | Nat8l | Ptger2 |
| Hoxc8 | Nccrp1 | Ptges |
| Hrc | Ncl | Ptgs2 |
| Hsd3b3 | Ndufb4 | Ptms |
| Hsdl2 | Nfe2 | Ptpdc1 |
| Hspa13 | Nhsl1 | Ptx3 |
| Hspa4 | Nmt2 | Pxn |
| Hspb6 | Nr2c1 | Rabl3 |
| Hspb7 | Nr2c2 | Rac2 |
| Hspd1 | Nrg1 | Rai14 |
| Hspg2 | Nrip1 | Rassf1 |
| Hvcn1 | Nt5dc2 | Raver1 |
| Ibsp | Nucks1 | Rbm9 |
| Icam2 | Nufip2 | Rbp1 |
| Ids | Nup133 | Reg3g |
| Ifi44 | Oasl1 | Rgs16 |
| Ifi47 | Obfc2a | Rgs2 |
| Ifit1 | Ociad2 | Rif1 |
| Ifit3 | Ogfod1 | Ripk3 |
| Ifnar2 | Oit1 | Rnf213 |
| Ift122 | Osbp2 | Rrad |
| Igf2r | Otud7b | Rras2 |
| Igh-6 | P2ry2 | Runx1 |
| Ighg | P4ha1 | Saa3 |
| Ighg1 | Padi3 | Samsn1 |
| Igj | Padi4 | Sass6 |
| Igk-V19-20 | Pah | Sat2 |
| Igl-V1 | Pak1 | Sell |
| Igsf5 /// Pcp4 | Palld | Selplg |
| Iigp1 | Papss2 | Serinc2 |
| Ikzf5 | Paqr8 | Serpina3g |
| Il10 | Pbx1 | Serpinb6b |
| Il10ra | Pcgf6 | Serpinb9 |
| Il11 | Pdlim5 | Serpine1 |
| Il13ra2 | Pdzk1 | Serpinh1 |
| Il21r | Pfkfb1 | Sez6l2 |
| Il33 | Pfkl | Sfrp2 |
| Ilk | Pfn1 | Sfrs6 |
| Inpp5d | Pgf | Sfrs7 |
| Inpp5j | Phf17 | Sgol2 |
| Invs | Phf3 | Sh2d5 |
| Iqgap2 | Phlda2 | Sh3kbp1 |
| Irf1 | Phldb2 | Sirpb1a |
| Irf2bp2 | Piga | Slc14a1 |
| Irf8 | Pign | Slc16a3 |
| Irgm2 | Pik3ap1 | Slc20a1 |
| Isl1 | Pitpnm3 | Slc2a3 |
| Itgb3 | Plat | Slc7a4 |
| Itpkb | Plch1 | Slc8a1 |
| Itsn1 | Plek2 | Slfn1 |
| Jun | Plekhh1 | Slfn2 |
| Kank2 | Plk1 | Slfn3 /// Slfn4 |
| Kat2b | Plk4 | Slpi |
| Kbtbd10 | Pmaip1 | Snap23 |
| Kcnab2 | Pola1 | Snord116 |
| Kcnk16 | Polr1a | Snrpf |
| Kmo | Polr1d | Socs3 |
| Krtap7-1 | Ppbp | Sorl1 |
| Lair1 | Ppp1r3b | Sox2 |
| Lama4 | Ppp3cb | Sprr2f |
| Laptm5 | Ppp5c | Sptlc2 |
| Layn | Prelid2 | Srgn |
| Lcp2 | Prkg2 | Srrm2 |
| Ldb3 | Prmt6 | St3gal1 |
| Lgals3bp | Prps2 | St3gal4 |
| Lgmn | Prss22 | Steap1 |
| Lhx6 | Prtn3 | Steap2 |
| Lilrb3 | Psca | Stfa2l1 |
| Limd2 | Psma6 | Sulf1 |
| Limk1 | Ptgfrn | Supv3l1 |
| Lmod2 | Pthlh | Syncrip |
| LOC100044115 /// Rin2 | Ptprr | Synpo2 |
| LOC100044885 /// Vsig4 | Ptprz1 | Syvn1 |
| LOC100045551 /// Ppm1d | Ptrf | Tbc1d1 |
| LOC100046044 /// Nr2f1 | Rab20 | Tcfec |
| LOC100046186 /// Ramp3 | Rab40b | Tes |
| LOC100047481 /// Sec24b | Rad18 | Tfpi2 |
| LOC100048247 | Rad51ap1 | Tgfb1 |
| LOC100048346 /// Usp18 | Rad54l | Tgfbr2 |
| Mup1 /// Mup10 /// Mup12 /// Mup13 | Rap2b | Timp1 |
| LOC432459 | Rapgef3 | Tm2d1 |
| LOC75771 | Rasgrf1 | Tmem106a |
| Loxl1 | Rbbp8 | Tmem217 |
| Lpar1 | Rbl1 | Tmsb10 |
| Lpxn | Rbm25 | Tnc |
| Lrp2bp | Rbms2 | Tnfaip2 |
| Lrrc2 | Rbp2 | Tnfrsf22 /// Tnfrsf23 |
| Lrrc33 | Rbpms | Tnfrsf23 |
| Lrrc8d | Rc3h2 | Tnfsf11 |
| Lst1 | Reep3 | Top2a |
| Ltbp1 | Rem2 | Tpm1 |
| Ltbp2 | Rg9mtd2 | Trem1 |
| Ly75 | Rnf122 | Trem3 |
| Ly86 | Rnf141 | Trim30 |
| Lyl1 | Rnf183 | Trmt11 |
| Macf1 | Rnpep | Tsix |
| Man1a | Rpe | Tubb6 |
| Man1c1 | Rpl14 | Txndc15 |
| Mapre3 | Rpl39l | Ube2c |
| Masp1 | Rrm1 | Upp1 |
| Matr3 | Rrm2 | Vcam1 |
| Mb | Rufy2 | Wdr65 |
| Mbd1 | Runx2 | Wisp1 |
| Mcpt2 | Ruvbl2 | Wnt5a |
| Mecp2 | Samd12 | Zfp719 |
| Med12l | Samhd1 |  |
| Mest | Scd2 |  |
| Mgat5 | Sdc1 |  |
| Mgp | Sdccag3 |  |
| Mme | Sec61a1 |  |
| Mocos | Sema4d |  |
| Mov10 | Serpinb9g |  |
| Mrc1 | Set |  |
| Mrc2 | Sfrs12 |  |
| Mrpl33 | Shmt2 |  |
| Mrps6 | Shroom3 |  |
| Ms4a6b | Slc13a1 |  |
| Ms4a6c | Slc16a1 |  |
| Msh5 | Slc26a4 |  |
| Msn | Slc27a2 |  |
| Msr1 | Slc38a4 |  |
| Msx3 | Slc4a1 |  |
| Mtap4 | Slc4a7 |  |
| Mterf | Slco1a5 |  |
| Mtss1 | Slco2a1 |  |
| Muc13 | Smg1 |  |
| Mx1 | Smox |  |
| Mybpc1 | Snai3 |  |
| Mybpc2 | Socs4 |  |
| Myct1 | Sorcs2 |  |
| Myf6 | Sox11 |  |
| Myh2 | Sox6 |  |
| Myh4 | Sp3 |  |
| Myl1 | Sparc |  |
| Mylpf | Speer7-ps1 /// Speer8-ps1 |  |
| Myom1 | Sprr1b |  |
| Myom2 | Sprr2h |  |
| Myot | Sprr2i |  |
| Myoz1 | Sprr2j-ps |  |
| Myoz2 | Sprr2k |  |
| Mypn | Sprr3 |  |
| Nav1 | St3gal6 |  |
| Nbea | Stk39 |  |
| Nceh1 | Stom |  |
| Ndst4 | Stra6 |  |
| Neto2 | Stx11 |  |
| Nhsl2 | Surf2 |  |
| Nid1 | Susd1 |  |
| Nisch | Swap70 |  |
| Nmur2 | Tbc1d9b |  |
| Nnt | Tbl1xr1 |  |
| Notch4 | Tceb3 |  |
| Npc2 | Tcerg1 |  |
| Nppb | Tcfap2e |  |
| Nr2f2 | Tctex1d2 |  |
| Nr4a2 | Tex15 |  |
| Nr4a3 | Tex19.1 |  |
| Nrap | Tgfbr1 |  |
| Nrp1 | Tmc7 |  |
| Nudt21 | Tmem183a |  |
| Oas1a | Tmem41b |  |
| Oas2 | Tmem47 |  |
| Odz3 | Tmigd1 |  |
| Ogt | Tmod3 |  |
| Olfml2b | Tmprss11d |  |
| Osr1 | Tmprss11f |  |
| P2ry14 | Tnfrsf12a |  |
| P2ry6 | Tnfrsf22 |  |
| Pabpc4l | Tnfsf9 |  |
| Parp14 | Tnik |  |
| Parvb | Tnks2 |  |
| Parvg | Tnnt2 |  |
| Pax6 | Tpd52 |  |
| Pcdh12 | Tpm3 |  |
| Pcdh18 | Traf4 |  |
| Pcdh19 | Troap |  |
| Pcdhg@ | Trp63 |  |
| Pcdhga1 /// Pcdhga10 /// Pcdhga11 /// | Tspan1 |  |
| Pck2 | Tspan6 |  |
| Pde4dip | Tspan9 |  |
| Pde7b | Ttc39b |  |
| Pdgfc | Tubb2a-ps2 /// Tubb2b |  |
| Pdgfra | Ube2s |  |
| Pdgfrb | Ube3a |  |
| Pdgfrl | Ubfd1 |  |
| Pdk4 | Ubl4 |  |
| Pdlim3 | Ubxn8 |  |
| Pdlim7 | Ufd1l |  |
| Pdxdc1 | Uhrf1 |  |
| Peg3 | Umod |  |
| Pgam2 | Usp27x |  |
| Phactr4 | Uxs1 |  |
| Phex | Vegfa |  |
| Phip | Vnn1 |  |
| Pi4k2a | Vps8 |  |
| Pif1 | Vsnl1 |  |
| Pik3cd | Wdr1 |  |
| Pik3ip1 | Wdr5 |  |
| Pitpnm1 | Wdr66 |  |
| Pkd2 | Wfdc2 |  |
| Pkn1 /// Ptger1 | Wnk1 |  |
| Pla2g15 | Wnk4 |  |
| Plek | Xiap |  |
| Plekho1 | Xpot |  |
| Plod1 | Yaf2 |  |
| Plxdc1 | Ywhag |  |
| Plxnc1 | Zbtb16 |  |
| Plxnd1 | Zfp106 |  |
| Pofut2 | Zfp462 |  |
| Pold3 | Zfp655 |  |
| Pou2af1 | Zxdc |  |
| Praf2 |  |  |
| Prdx3 |  |  |
| Prkca |  |  |
| Prkd1 |  |  |
| Prl3d1 |  |  |
| Prl3d1 /// Prl3d2 |  |  |
| Prnd |  |  |
| Prrg1 |  |  |
| Prune2 |  |  |
| Psip1 |  |  |
| Ptgfr |  |  |
| Ptgs1 |  |  |
| Ptplad2 |  |  |
| Ptprc |  |  |
| Ptprs |  |  |
| Pvalb |  |  |
| Pvr |  |  |
| Pvt1 |  |  |
| Pycr1 |  |  |
| Pygm |  |  |
| Rab31 |  |  |
| Rab3il1 |  |  |
| Ralb |  |  |
| Rap1b |  |  |
| Raph1 |  |  |
| Rarres2 |  |  |
| Rasa1 |  |  |
| Rbbp4 |  |  |
| Rbm26 |  |  |
| Rbms3 |  |  |
| Rbpj |  |  |
| Reg1 |  |  |
| Retnla |  |  |
| Rffl |  |  |
| Rgs1 |  |  |
| Rgs10 |  |  |
| Rhoc |  |  |
| Rictor |  |  |
| Rptor |  |  |
| Rrbp1 |  |  |
| Rtn2 |  |  |
| Rtp4 |  |  |
| Runx3 |  |  |
| Ryr1 |  |  |
| S1pr3 |  |  |
| Saa1 |  |  |
| Saa2 |  |  |
| Safb2 |  |  |
| Samd5 |  |  |
| Sbno2 |  |  |
| Scfd2 |  |  |
| Schip1 |  |  |
| Sdc2 |  |  |
| Sec16b |  |  |
| Sema3a |  |  |
| Sema5a |  |  |
| Sema6d |  |  |
| Sema7a |  |  |
| Sept6 |  |  |
| Sept9 |  |  |
| Serf2 |  |  |
| Serpina1a /// Serpina1b |  |  |
| Serpina1b |  |  |
| Serpinb12 |  |  |
| Sestd1 |  |  |
| Sf3b2 |  |  |
| Sfpi1 |  |  |
| Sfxn5 |  |  |
| Shisa4 |  |  |
| Sidt2 |  |  |
| Sipa1 |  |  |
| Sirpa |  |  |
| Sirt4 |  |  |
| Six4 |  |  |
| Skp2 |  |  |
| Sla |  |  |
| Slc11a1 |  |  |
| Slc15a3 |  |  |
| Slc25a24 |  |  |
| Slc25a30 |  |  |
| Slc38a1 |  |  |
| Slc39a14 |  |  |
| Slc41a2 |  |  |
| Slc46a1 |  |  |
| Slc5a3 |  |  |
| Slc7a7 |  |  |
| Slc8a2 |  |  |
| Slc8a3 |  |  |
| Slc9a8 |  |  |
| Slfn5 |  |  |
| Slfn8 |  |  |
| Sln |  |  |
| Sms |  |  |
| Snai1 |  |  |
| Snx5 |  |  |
| Socs5 |  |  |
| Sox18 |  |  |
| Sox4 |  |  |
| Sp100 |  |  |
| Spata1 |  |  |
| Spef1 |  |  |
| Spp1 |  |  |
| Spred3 |  |  |
| Spry4 |  |  |
| Spsb1 |  |  |
| Srl |  |  |
| St3gal2 |  |  |
| St6galnac4 |  |  |
| St8sia4 |  |  |
| Stab1 |  |  |
| Stard9 |  |  |
| Stat1 |  |  |
| Stat4 |  |  |
| Stc1 |  |  |
| Stk32b |  |  |
| Stx18 |  |  |
| Stx2 |  |  |
| Styx |  |  |
| Sulf2 |  |  |
| Sykb |  |  |
| Syne1 |  |  |
| Synpo2l |  |  |
| Tardbp |  |  |
| Tcap |  |  |
| Tfpi |  |  |
| Tgfb1i1 |  |  |
| Tgm2 |  |  |
| Tgtp1 /// Tgtp2 |  |  |
| Thbs4 |  |  |
| Thsd4 |  |  |
| Tifab |  |  |
| Tln1 |  |  |
| Tlr1 |  |  |
| Tlr2 |  |  |
| Tlr4 |  |  |
| Tm4sf1 |  |  |
| Tm6sf1 |  |  |
| Tmcc2 |  |  |
| Tmem107 |  |  |
| Tmem173 |  |  |
| Tmem56 |  |  |
| Tmem74 |  |  |
| Tmem8 |  |  |
| Tmem86b |  |  |
| Tmem88 |  |  |
| Tmod4 |  |  |
| Tnfaip6 |  |  |
| Tnfaip8l2 |  |  |
| Tnfrsf11b |  |  |
| Tnfrsf1b |  |  |
| Tnfrsf9 |  |  |
| Tnnc2 |  |  |
| Tnni2 |  |  |
| Tnnt3 |  |  |
| Tnrc6a |  |  |
| Tomm7 |  |  |
| Tor3a |  |  |
| Tpm2 |  |  |
| Traf1 |  |  |
| Traf2 |  |  |
| Trim63 |  |  |
| Trp53 |  |  |
| Trp53tg5 |  |  |
| Trpm6 |  |  |
| Trpv2 |  |  |
| Tsc22d1 |  |  |
| Tspan11 |  |  |
| Tspan4 |  |  |
| Tspan8 |  |  |
| Ttc21a |  |  |
| Ttn |  |  |
| Ttyh1 |  |  |
| Tubb5 |  |  |
| Tubgcp4 |  |  |
| Tulp3 |  |  |
| Twf2 |  |  |
| Tyrobp |  |  |
| Uap1l1 |  |  |
| Ube2l3 |  |  |
| Ubxn11 |  |  |
| Unc13c |  |  |
| Unc93b1 |  |  |
| Urgcp |  |  |
| Ushbp1 |  |  |
| Vasp |  |  |
| Vav1 |  |  |
| Vcan |  |  |
| Vldlr |  |  |
| Vps13a |  |  |
| Was |  |  |
| Wasf1 |  |  |
| Wtip |  |  |
| Zar1 |  |  |
| Zbtb32 |  |  |
| Zbtb8os |  |  |
| Zeb1 |  |  |
| Zeb2 |  |  |
| Zfhx3 |  |  |
| Zfp281 |  |  |
| Zfp827 |  |  |
| Zfp93 |  |  |
| Zfpm2 |  |  |
| Zmynd15 |  |  |
| Zzz3 |  |  |
