# Supplementary material for: Heterogeneity of Gene Expression in Murine Squamous Cell Carcinoma Development—The Same Tumor by Different Means
Source: PLoS One. 2013 Mar 18;8(3):e57748. doi: 10.1371/journal.pone.0057748 (PMC3601100; doi:10.1371/journal.pone.0057748)
Supplement: Table S3 — Genes that were increased in at least 4-fold change and were involved in “Cytokine-cytokine receptor interaction” according to KEGG in mouse ID7 and mouse ID12. (DOCX) [file pone.0057748.s003.docx]

**Table S3**

| **Genes increased in Mouse ID7 (21)** | **Genes increased in Mouse ID12 (9)** | **Genes increased in both mice (27)** |
| --- | --- | --- |
| Ccl11 | Cxcl9 | Ccl12 |
| Ccl24 | Il1a | Ccl3 |
| Ccl4 | Il1rap | Ccl8 |
| Ccl5 | Il23a | Ccl9 |
| Ccl6 | Ppbp | Ccr1 |
| Ccl7 | Tgfbr1 | Ccr5 |
| Csf1r | Tnfrsf12a | Cd40 |
| Csf2ra | Tnfsf9 | Clcf1 |
| Flt1 | Vegfa | Csf3 |
| Hgf |  | Csf3r |
| Ifnar2 |  | Cxcl1 |
| Il10 |  | Cxcl16 |
| Il10ra |  | Cxcl2 |
| Il11 |  | Cxcl5 |
| Il21r |  | Cxcr2 |
| Pdgfc |  | Cxcr4 |
| Pdgfra |  | Il18rap |
| Pdgfrb |  | Il1b |
| Tnfrsf11b |  | Il2rg |
| Tnfrsf1b |  | Il4ra |
| Tnfrsf9 |  | Il6 |
|  |  | Inhba |
|  |  | Met |
|  |  | Osm |
|  |  | Tgfb1 |
|  |  | Tgfbr2 |
|  |  | Tnfsf11 |
