# Supplementary material for: Heterogeneity of Gene Expression in Murine Squamous Cell Carcinoma Development—The Same Tumor by Different Means
Source: PLoS One. 2013 Mar 18;8(3):e57748. doi: 10.1371/journal.pone.0057748 (PMC3601100; doi:10.1371/journal.pone.0057748)
Supplement: Table S4 — Genes that were increased in at least 4-fold change and were involved in “Metabolic pathways” according to KEGG in mouse ID7 and mouse ID12. (DOCX) [file pone.0057748.s004.docx]

**Table S4**

| **Genes increased in Mouse ID7 (45)** | **Genes increased in Mouse ID12 (45)** | **Genes increased in both mice (28)** |
| --- | --- | --- |
| Acsl3 | Acadl | Ak1 |
| Adssl1 | Acsl4 | Auh |
| Agmat | Ahcy | B4galnt1 |
| Agps | Akr1b3 | B4galt6 |
| Ahcyl2 | Akr1b8 | Bcat1 |
| Aldh18a1 | Aldh1a3 | Bst1 |
| Atp6v0d2 | Aldh7a1 | Cyp4f18 |
| B3galnt1 | Arg1 | Enpp1 |
| Ckm | Atp5h | Ept1 |
| Cox6a2 | B3gnt3 | Etnk1 |
| Cox8b | Bst1 | Gcnt1 |
| Csgalnact1 | C1galt1 | Gcnt2 |
| Cyp2c65 | Degs2 | Gfpt1 |
| Cyp4f16 | Dgka | Hdc |
| Eno1 | Galnt3 | Hk3 |
| Eno3 | Galnt7 | Lclat1 |
| Ext1 | Glb1 | Lipg |
| Fut8 | Gmppb | Lpcat1 |
| Galk1 | Gnpda2 | Mgat3 |
| Galnt10 | Hpgds | Mtap |
| Galnt2 | Lpcat4 | Nans |
| Ganc | Lpin2 | Ptges |
| Gm5506 | Mat2a | Ptgs2 |
| Gusb | Mboat1 | Sat2 |
| hexosaminidase A | Mgam | Sptlc2 |
| Hmgcr | Mthfd2 | St3gal1 |
| Ids | Mtm1 | St3gal4 |
| Inpp5j | Ndufb4 | Upp1 |
| Itpkb | Odc1 |  |
| Kmo | P4ha1 |  |
| Man1a | Pah |  |
| Man1c1 | Papss2 |  |
| Mgat5 | Pfkl |  |
| Ndst4 | Piga |  |
| Nnt | Pign |  |
| Pck2 | Pola1 |  |
| Pgam2 | Polr1a |  |
| Pi4k2a | Polr1d |  |
| Pold3 | Prps2 |  |
| Ptgs1 | Rpe |  |
| Pycr1 | Rrm1 |  |
| Sms | Rrm2 |  |
| St3gal2 | Shmt2 |  |
| St6galnac4 | St3gal6 |  |
| Uap1l1 | Uxs1 |  |
