# Supplementary material for: Heterogeneity of Gene Expression in Murine Squamous Cell Carcinoma Development—The Same Tumor by Different Means
Source: PLoS One. 2013 Mar 18;8(3):e57748. doi: 10.1371/journal.pone.0057748 (PMC3601100; doi:10.1371/journal.pone.0057748)
Supplement: Table S5 — Genes that were decreased in at least 4-fold change and were involved in “Metabolic pathways” according to KEGG in mouse ID7 and mouse ID12. (DOCX) [file pone.0057748.s005.docx]

**Table S5**

| **Genes decreased in Mouse ID7 (37)** | **Genes decreased in Mouse ID12 (17)** | **Genes decreased in both mice (35)** |
| --- | --- | --- |
| Acsbg1 | Agpat4 | Acer1 |
| Acsl1 | Aldh6a1 | Acox2 |
| Aldh3b2 | Atp6v0a4 | Adh1 |
| Aldoc | Atp6v0e2 | Adh7 |
| Alox12b | B4galt7 | Aldh1a7 |
| Atp6v0a1 | Dct | Aldh3a1 |
| Bdh1 | Gal3st1 | Alox8 |
| Cds1 | Gpam | Amy1 |
| Ckmt1 | Hal | Cyp1a1 |
| Cyp2e1 | Hkdc1 | Cyp2b10 |
| Cyp2j11 | Hlcs | Cyp2b19 |
| Cyp2j6 | Ids | Cyp8b1 |
| Cyp3a13 | Ppap2b | Dbt |
| Cyp4f18 | Ptgds | Eprs |
| Cyp4f39 | Ptgis | Fbp1 |
| Dgat2 | Sgms1 | Haao |
| Dhcr24 | Sphk1 | Hsd3b2 |
| Extl3 |  | Hsd3b6 |
| Fdft1 |  | Lass4 |
| Glt28d2 |  | Mboat2 |
| Hmgcs2 |  | Me1 |
| Hsd17b12 |  | Mgll |
| Hsd17b2 |  | Olah |
| Idi1 |  | Pank1 |
| Lass3 |  | Pigh |
| Lass4 |  | Pla2g2e |
| Lpin3 |  | Pla2g5 |
| Ndufs6 |  | Rdh1 |
| Nt5c3 |  | Rdh11 |
| Pla2g4b |  | Rgn |
| Rdh11 |  | Scp2 |
| Rdh12 |  | Smpd3 |
| Rdh16 |  | Sms |
| Sc4mol |  | Sptlc3 |
| Sgms2 |  | Tyrp1 |
| Sptlc1 |  |  |
| Tst |  |  |
